# Molecular genetic variability of *Cryphonectria hypovirus* 1 associated with *Cryphonectria parasitica* in South Tyrol (northern Italy)

**DOI:** 10.3389/fmicb.2024.1291542

**Published:** 2024-02-27

**Authors:** Farooq Ahmad, Selena Tomada, Thanalai Poonsiri, Sanja Baric

**Affiliations:** ^1^Laboratory for Phytopathology, Faculty of Agricultural, Environmental and Food Sciences, Free University of Bozen-Bolzano, Bolzano, Italy; ^2^Department of Pathobiology, Faculty of Science, Mahidol University, Bangkok, Thailand; ^3^Competence Centre for Plant Health, Free University of Bozen-Bolzano, Bolzano, Italy

**Keywords:** dsRNA viruses, mycoviruses, hypovirulence, immunoassay, RT-PCR, chestnut blight

## Abstract

*Cryphonectria hypovirus* 1 (CHV-1) has been widely studied and used as a biocontrol agent because of its ability to infect the chestnut blight fungus, *Cryphonectria parasitica*, and to reduce its virulence. Knowledge about the hypovirus, its presence, and diversity is completely lacking in South Tyrol (northern Italy), which may obstruct biocontrol measures for chestnut blight based on CHV-1. This work aimed to study the occurrence of CHV-1 infecting *C. parasitica* in South Tyrol and to perform a genetic characterization of the hypovirus. In South Tyrol, CHV-1 was found to occur in 29.2% of the fungal isolates investigated, varying in frequency between different regions and chestnut stands. Twenty-three haplotypes based on partial cDNA (complementary DNA) sequences of open reading frame (ORF)-A and 30 haplotypes based on partial cDNA sequences of ORF-B were identified among 47 and 56 hypovirulent fungal isolates, respectively. Phylogenetic analysis showed that all the haplotypes belonged to the Italian subtype of CHV-1 and that they were closely related to the populations of Italy, Switzerland, Croatia and Slovenia. Evidence of recombination was not found in the sequences and point mutations were the main source of diversity. Overall, this study indicated that the prevalence of CHV-1 in South Tyrol is low compared to many other central and western European populations and determined a need to actively impose biocontrol measures. Using sequence analysis, we identified some variants of interest of CHV-1 that should be studied in detail for their potential use in biocontrol.

## 1 Introduction

Mycoviruses infect fungi and cause considerable changes in their phenotype, such as discoloration, abnormal growth and decreased sporulation (Nuss, 2005). Some mycoviruses can reduce virulence and pathogenicity of plant pathogenic fungi, referred to as hypovirulence (Choi and Nuss, 1992; Heiniger and Rigling, 2009). Consequently, mycoviruses have caught particular attention for practical applications as biocontrol agents against pathogenic fungi, as reviewed by Heiniger and Rigling (2009). Hypoviruses are positive-stranded RNA viruses without a coat protein located in the cytoplasm of the fungal hosts (Suzuki et al., 2018). They were initially thought to be double-stranded (ds) RNA viruses, which later was found to be the replicative intermediate form of hypoviruses (Suzuki et al., 2018). These viruses were previously a member of the genus *Hypovirus* and consisted of four characterized species: *Cryphonectria hypovirus* 1 (CHV-1), CHV-2, CHV-3 and CHV-4 (Hillman and Suzuki, 2004; Turina and Rostagno, 2007). However, the nomenclature of these viruses has recently been updated. The old genus *Hypovirus* was split to make *Alphahypovirus* and *Betahypovirus*. Therefore, CHV-1 belongs to the species *Alphahypovirus cryphonectriae* (Chiba et al., 2023). The most common host associated with *Hypovirus* is *Cryphonectria parasitica* (Murrill) Barr (Suzuki et al., 2018), although some unclassified hypoviruses are known to infect other fungal species (Khalifa and Pearson, 2014; Li et al., 2019.

*Cryphonectria parasitica* is an ascomycete fungus responsible for a devastating disease of chestnut trees (*Castanea* spp.) called chestnut blight, which is relevant from an ecological and economical point of view. The fungus infects the bark of branches and stems and causes bark cankers. The cankers can lead to the wilting of plant organs above the girdling site and to the death of infected trees. *Cryphonectria parasitica* is the most important pathogen of chestnut trees that caused the loss of approximately four billion chestnut trees in North America (Roane et al., 1986). It was first detected in New York at the beginning of the 20^th^ century (Anagnostakis, 1987). In Europe, it was detected in 1938 near the port of Genoa in north-western Italy (Biraghi, 1946). From northern Italy, *C. parasitica* successfully spread all over the European continent and is now present in almost all chestnut-growing regions of Europe (Rigling and Prospero, 2018). The disease in Europe is not as severe as in North America because of the manifestation of superficial cankers, which generally do not lead to the dieback of the infected trees (Heiniger and Rigling, 1994). Previously thought of as the intrinsic resistance of European chestnut (*Castanea sativa*), this feature was later identified as a viral infection of *C. parasitica* by hypoviruses inducing hypovirulence (Anagnostakis, 1987).

The *Cryphonectria-Hypovirus* pathosystem is a famous example of biocontrol, which has been quite successfully applied in Europe (Heiniger and Rigling, 1994; Milgroom and Cortesi, 2004). CHV-1 is the most important virus that infects *C. parasitica*, which can be transmitted horizontally through hyphal anastomosis among vegetative compatible (VC) fungal strains and vertically (from one generation to another) through asexual spores (Rigling and Prospero, 2018). Virus-containing *C. parasitica* strains, referred to as hypovirulent, are currently present in many chestnut-growing areas of Europe, either naturally or as a result of artificial introductions (Sotirovski et al., 2006; Liu et al., 2007; Krstin et al., 2011, 2020; Peters et al., 2014; Rigling et al., 2018). However, virus-free (virulent) strains of *C. parasitica* continue to cause economic losses and a high mortality rate of chestnut trees is still observed in different regions of Europe, probably because of a combined effect of the pathogen and changing climate, as an association study found higher mortality rates in drought-affected diseased trees (Waldboth and Oberhuber, 2009).

Knowledge about the presence of hypoviruses in fungal strains and their frequency in a population is essential to monitor a mycovirus-mediated biocontrol program and to take plant protection measures accordingly. As some viruses induce changes in the appearance of fungi, the traditional method to detect the presence of mycoviruses was based on morphology (Zamora et al., 2007). Virulent strains of *C. parasitica* generally display a characteristic dark yellow to orange colored mycelium on culture media and form a high number of pycnidia carrying mature conidia (Rigling and Prospero, 2018). CHV-1 infections, in turn, often cause mycelium discoloration, whereas sporulation is largely reduced to almost none (Aguin et al., 2005). However, not all hypoviruses induce morphological changes in fungi, or the changes are not always easy to detect, mainly because of intermediate morphology (Krstin et al., 2008; Castaño et al., 2014). This led to the development of methods for the molecular determination of mycoviruses. One approach is to isolate dsRNA and to separate and visualize it directly on agarose gels; however, the instability of RNA and low amounts of dsRNA present in *C. parasitica* tend to give false-negative results (Zamora et al., 2007). The application of reverse transcription-polymerase chain reaction (RT-PCR) in combination with agarose gel electrophoresis represented the next step in the evolution of hypovirus detection. This method is quite accurate for detecting mycoviruses but is time-consuming and relatively expensive, making it impractical for larger sample sizes. Still, RT-PCR complemented with Sanger sequencing is largely used to study population genetics and variability of mycoviruses (Gobbin et al., 2003; Liu et al., 2007; Krstin et al., 2008, 2020; Sotirovski et al., 2011; Zamora et al., 2012; Castaño et al., 2014; Rigling et al., 2018; Del Carratore et al., 2021). However, due to cost and time issues, the sample size in some population genetic studies of mycoviruses was significantly reduced as compared to the fungal host (Krstin et al., 2008, 2011) or relied on phenotypic screening to select the isolates for further molecular analysis (Bissegger et al., 1997; Zamora et al., 2012; Castaño et al., 2014). Immunological methods, such as enzyme-linked immunosorbent assay (ELISA), have been successfully applied for several decades to detect human, animal, and plant viruses (Aramburu and Moreno, 1994; Peruski and Peruski, 2003), whereas tissue-blot immunoassays (TBIA) that detect pathogens directly from plant tissue imprints on nitrocellulose membranes proved very useful in the rapid detection of plant viruses (Djelouah et al., 2014). Yaegashi et al. (2011) developed a dot-blot immunoassay for the detection of mycoviruses with dsRNA genomes in *Rosellinia necatrix*, which, however, was never used in the *Cryphonectria-Hypovirus* system. Such a simple, rapid and inexpensive method based on the detection of dsRNA would be convenient to screen a large number of fungal isolates for the presence of mycoviruses and would therefore represent a useful tool to accompany biocontrol measures.

After detecting the presence of mycoviruses, it may be required to determine the types of mycoviruses present in a population. For example, in *C. parasitica*, different subtypes of CHV-1 were identified based on the sequence variation in the open reading frame A (ORF-A) (Gobbin et al., 2003). Subtype I (Italian) is the most common and widespread subtype of CHV-1 in several European countries such as Bosnia-Herzegovina, Croatia, south-eastern France, Greece, Italy, Macedonia, Slovenia, Switzerland and Turkey (Allemann et al., 1999; Sotirovski et al., 2006; Krstin et al., 2011; Akilli et al., 2013). Other subtypes (such as F1, F2, D, E, and G) have been found mainly in France, Germany, Spain, Eastern Turkey and Georgia (Zamora et al., 2012; Akilli et al., 2013; Feau et al., 2014; Peters et al., 2014; Rigling et al., 2018). Information about the geographical distribution of these subtypes matches well with the distribution of *C. parasitica*, which suggests multiple introduction events of the fungus and CHV-1 (Rigling and Prospero, 2018). The subtypes also differed in their degree of hypovirulence and overall effects on the fungal host (Morozov et al., 2007; Robin et al., 2010). Subtype I, for example, has a milder effect on the host; hence, the virus can establish itself better in a fungal population. In contrast, the French subtypes (F1 and F2) severely impact the growth and sporulation of its fungal host and hence limit the spread of hypovirulence in a population (Robin et al., 2010). Within subtype I, some mutations in the ORF might change the effect of CHV-1 on the phenotype of its fungal host (Krstin et al., 2020). Changes in the virulence of subtype I were found in many regions, for example, UK (Romon-Ochoa et al., 2020), Croatia (Nuskern et al., 2017; Krstin et al., 2020) and Macedonia (Sotirovski et al., 2011), which could also be due to mutations in the ORFs. Although the genome of a central Italian CHV-1 strain was recently characterized (Murolo et al., 2018), it is unknown which kind of mutations happened in the North Italian populations of CHV-1 as no data is available about its genetic diversity within the region.

Chestnut trees in South Tyrol (northern Italy) are important to the local landscape and forests. Two hundred and sixty-two chestnut stands were revealed in the region in the last census of agriculture in 2010, which contribute additional income to farmers and promote tourism (Fedrigotti and Fischer, 2015). However, a high mortality rate of chestnut trees has been observed in South Tyrol, probably caused by a combined effect of climate change, *C. parasitica* infection and loss of hypovirulence (Waldboth and Oberhuber, 2009). *Cryphonectria parasitica* populations in South Tyrol were recently characterized based on molecular genetic markers, which pointed to a high degree of diversity and variability of the fungus (Ahmad and Baric, 2022a,b). CHV-1 depends on *C. parasitica* for migration among sub-populations; therefore, a high genetic diversity of *C. parasitica* gives a first indication of the genetic variation among the virus, as it was observed that the evolution of CHV-1 is spatially congruent with the evolution of *C. parasitica* (Bryner et al., 2012). Thus, the aims of the present study were the following: (1) To detect the presence and occurrence of dsRNA infecting *C. parasitica* in South Tyrol through morphological, immunological and molecular genetic methods; (2) To investigate the molecular genetic variability of CHV-1 present in South Tyrol by analyzing nucleic acid sequence variation and comparing it with sequences from other regions to understand its evolution; (3) To study the conservation of amino acids in the sequences of CHV-1 present in South Tyrol in order to infer the possible functioning of CHV-1 polyproteins in the local isolates through *in-silico* and modeling analyses; (4) To identify naturally occurring strains of CHV-1 for their potential use in biocontrol based on the population genetic data and *in-silico* analyses of the mutations found in the ORFs fragments.

## 2 Materials and methods

### 2.1 Sampling and isolation of *Cryphonectria parasitica*

Chestnut bark samples were collected from trees showing characteristic symptoms of chestnut blight. Sampling was performed in all chestnut growing areas of South Tyrol (northern Italy) as previously described by Ahmad and Baric (2022a). A total of 263 isolates of *C. parasitica* were investigated in this study. The identity of the isolates was confirmed by phenotypic traits and molecular methods (Ahmad and Baric, 2022a). Details about isolates and their sampling locations can be found in Supplementary Table 1 and Supplementary Figure 1.

### 2.2 Phenotypic and immunological characterization of *Cryphonectria parasitica* for hypovirus presence

Fresh cultures of *C. parasitica* were grown on potato dextrose agar (PDA) at room temperature for phenotypic characterization (Bissegger et al., 1997). Pigmentation of the mycelium was observed visually after 1, 2, and 3 weeks of incubation, to assign the status of hypovirulence (Bissegger et al., 1997). Isolates were regarded as hypovirulent when the pigment was absent, as intermediate if a yellowish or light-orange pigment was present, and as virulent if the pigmentation was orange to dark orange (Murolo et al., 2018).

To detect the presence of dsRNA, a dot-blot immunoassay described by Yaegashi et al. (2011) based on an antigen-antibody reaction was adapted (Peever et al., 1998). Briefly, fresh cultures of *C. parasitica* strains were prepared on PDA and incubated at room temperature for 14 days. The fungal mycelium was removed with a sterile tip (avoiding transferring PDA) and placed in a 1.5 ml microcentrifuge tube containing 500 μl of a resuspension buffer (50 mM Tris-HCl, pH 7.5; 10 mM EDTA; 1% SDS). A 3-mm tungsten bead (Qiagen GmbH, Hilden, Germany) was added to the 1.5 ml tube, then vigorously mixed in a TissueLyser (Qiagen) for 2 min at a frequency of 30 Hz and left to rest for 1 min on ice. Protran™ Premium Western Blotting Nitrocellulose Membranes (Cytiva Amersham, United Kingdom) were cut into rectangles of approximately 4 × 1.5 cm and were labeled with a permanent marker (Faber-Castell, Germany). Ten microliter of crude extract were applied in triplicates on each piece of membrane and air-dried completely for at least 5 min. The membrane strips were incubated in a glass beaker containing hydrogen peroxide (6%) in Tween Tris Buffered Saline (TTBS) (20 mM Tris–HCl, 150 mM NaCl, 0.1% Tween 20; pH 7.5) at 50°C for 60 min on a hot-plate with stirring. The membrane strips were washed with TTBS three times at room temperature and blocked with skim milk (5%) in TTBS at 30°C for 60 min. The membrane strips were then incubated with a dsRNA-specific monoclonal antibody, J2 (Scion, Budapest, Hungary), diluted 1:2,500 in TTBS at 5°C for 14–18 h with gentle stirring. The membrane strips were washed three times with TTBS and incubated in TTBS treated with anti-mouse IgG horseradish-peroxidase (HRP)-linked secondary antibody (Sigma-Aldrich, Germany) (1:2,500) at 30°C for 60 min. The membranes were washed three times again with TTBS. Ten microliter Chromogenic HRP Enzyme Substrate TMB (3,3′,5,5′-tetramethylbenzidine; Bio-Rad, Hercules, CA, USA) were applied on the positions of each sample replicate on the membrane strips. After 5–10 min of drying, color change was observed to detect the antigen-antibody reaction. A positive reaction was characterized by at least one replicate showing a greenish-blue color dot. In contrast, a negative reaction was characterized by no color change in all triplicates (white membrane).

### 2.3 RT-PCR and Sanger sequencing of amplified ORF-A and ORF-B fragments of CHV-1

The *C. parasitica* isolates, which tested positive in the immunological assay, were subjected to further molecular analysis. The isolates were grown on PDA covered with a sterile cellophane membrane for 7–10 days at room temperature in the dark. Total RNA was extracted using Plant/Fungi Total RNA purification kit manufactured by Norgen Biotek Corporation (Thorold, Canada), following the manufacturer's instructions. Reverse transcription (RT) of the extracted RNA was performed using the First Strand cDNA Synthesis kit (Thermo Fisher, Vilnius, Lithuania), following the manufacturer's protocol. Polymerase chain reaction (PCR) was performed using cDNA as a template. Two loci of CHV-1 were chosen to be amplified that were located in ORF-A and ORF-B (Allemann et al., 1999; Gobbin et al., 2003). PCR amplification was carried out as following: 10 μl of 2 × PCRBIO HS Taq Mix (PCR Biosystems, London, United Kingdom), 1 μl of cDNA, 0.25 μl of the respective primers (10 μM; Supplementary Table 2), and nuclease-free water to adjust the final volume of 20 μl. The PCR conditions were the following: denaturation at 95°C for 2 min followed by 38 cycles (ORF-A) or 33 cycles (ORF-B) of 95°C for 30 s, 52.5°C (ORF-A) or 55°C (ORF-B) of annealing for 1 min and 30 s, and 72°C for 2 min. The final extension was done at 72°C for 8 min. The amplicons were separated on agarose gels (1.5%) stained with MIDORI Green (Nippon Genetics, Saitama, Japan) and visualized on a gel documentation system. Beside all the *C. parasitica* isolates, which tested positive in the dot-blot immunoassay, 26 (14.4%) of the negatively tested samples were randomly selected and tested with RT-PCR. This was done to estimate false positive and false negative rates by using RT-PCR as the reference standard. The final dataset about the occurrence of CHV-1 in South Tyrol was based on the results obtained from the RT-PCR assay.

Sanger sequencing of ORF-A and ORF-B fragments was performed of all amplified isolates to study the genetic diversity of the CHV-1 present in South Tyrol. PCR products were purified using the enzymatic PCR cleanup A'SAP (ArcticZymes, Tromsø, Norway). Primers Hvep-1 and Hvep-2 were used to sequence ORF-A fragment, whereas ORF-B was sequenced with amplification primers EP713-6 and EP713-7 (Supplementary Table 2). Private/rare haplotypes were confirmed by additional sequencing reactions using ORF-A fragment PCR primers EP713-5 and R2280 (Supplementary Table 2). Sequencing reactions and capillary electrophoresis were performed by Microsynth Seqlab GmbH (Göttingen, Germany).

### 2.4 Data analysis

Sequences obtained with forward and reverse primers (after generation of reverse-complements of the latter) for each isolate were assembled in the software Geneious Prime, version 2021.0.3 (Biomatters Ltd., Auckland, New Zealand) using the feature of *de-novo* assembly. Consensus sequences of each hypovirus strain were aligned using the Muscle alignment function implemented in Geneious Prime. The sequences were trimmed at both ends and sequences that were shorter than 1,202 bp (in case of ORF-A fragment) or 1,194 bp (in case of ORF-B fragment) were removed from the analysis. DnaSP (version 6) was used to calculate the number of haplotypes, haplotype diversity, nucleotide diversity and the average number of nucleotide differences per site among sequences (Rozas et al., 2017). The haplotypes were named HA/HB/HAB based on the ORF they represented; for example, HA1, HB1 and HAB1 were the first haplotypes of ORF-A, ORF-B fragments and combined sequences of ORF-A and B, respectively. The occurrence of recombination within the sequences of ORF-A and ORF-B was tested using RDP4 version 4.101 (Martin et al., 2015). Reference sequences for ORF-A and ORF-B fragments were downloaded from NCBI GenBank and aligned with sequences of local isolates. As for ORF-A, sequences with different lengths were available in GenBank, two thresholds were defined to obtain alignments of ORF-A with different lengths. Specifically, 84 long sequences obtained from GenBank were aligned with the sequences obtained in the present study to obtain an alignment with 1,160 bp. Three hundred and thirty-two short sequences from GenBank (different from the previous set) were downloaded from GenBank and aligned with our sequences to obtain an alignment of 561 bp. Haplotype networks were constructed using POPART software (version 1.7) through the median-joining and minimum-spanning network algorithms (Bandelt et al., 1999). Maximum likelihood trees were constructed in MEGA-X (version 10.0.5) with 1,000 bootstrap replications using the Tamura-Nei model (Tamura and Nei, 1993; Kumar et al., 2018).

Translation of aligned nucleic acid sequences into amino acid sequences was also performed with Geneious Prime version 2021.0.3. The translation reading frame was defined according to a representative strain of CHV-1 subtype I, EP721 (NCBI GenBank accession number: DQ861913) by aligning our sequences with it and translating the sequences according to its start codon. Amino acid conservation was calculated based on the physico-chemical properties of the alignments for ORF-A and ORF-B using Jalview (version 2.11.1.4). A numerical index from Jalview classifies the lowest conservation as 0 and the highest conservation as 11 (Waterhouse et al., 2009). Amino acid identity agreement with the consensus was also calculated through Jalview. All amino acid substitutions of ORF-A were also checked for their hypothetical effect on protein functioning through the web application SIFT (Sim et al., 2012). SIFT sorts amino acid substitutions into tolerated or deleterious (intolerant) based on the evolutionary properties of the protein using sequence identity (Ng and Henikoff, 2001). The number of total synonymous and non-synonymous polymorphic sites was calculated through DnaSP (Rozas et al., 2017). All sequences were checked against each other for the K_a_/K_s_ values in TBtools (Chen et al., 2020). K_a_/K_s_ is an indicator of selection pressure at the sequence level and its values represent the estimation of positive selection (K_a_/K_s_ > 1), negative selection (K_a_/K_s_ < 1) and neutral selection (K_a_/K_s_ = 1). Mean value (μ), standard deviation (SD) and standard error (SE) were determined of the whole K_a_/K_s_ data in RStudio (version 2023.12.0+369). A *Z*-test was performed to assess the statistical significance of each K_a_/K_s_ value that was higher than 1 (indicating positive selection) compared to the null hypothesis (K_a_/K_s_ = 1 or neutral selection). The *p*-values for each *Z*-score were obtained using a standard normal distribution table for a two-tailed test. Observations with *p*-values < 0.05 were considered statistically significant, leading to the rejection of the null hypothesis. Scatterplots of K_a_/K_s_ values were plotted in RStudio using ggplot2 library (Wickham, 2016).

A combined approach based on K_a_/K_s_ ratios and intolerant amino acid substitutions was used to identify variants of interest (VOIs). First, haplotypes were screened based on K_a_/K_s_ value (cutoff value ≤ 1). Haplotypes that had both a high probability of positive selection (K_a_/K_s_ >1) and presence of intolerant amino acids were included in the list of VOIs. Known functional sites that are important for the autocatalytic cleavage of ORF-A and ORF-B were manually checked for non-synonymous mutations to find out if some hypoviruses have potentially lost the proper functioning of these polyproteins (Krstin et al., 2020). Physico-chemical distances among mutations of interest were also calculated through Grantham's distance matrix (Grantham, 1974). The Grantham score is a numerical approach used to calculate dissimilarity between amino acids based on three physico-chemical properties i.e., composition, polarity and molecular volume (Grantham, 1974). Based on Grantham scores, non-synonymous substitutions were designated as conservative (0–50), moderately conservative (51–100), moderately radical (101–150) and radical (>151) as proposed by Li et al. (1984).

The full-length sequences of CHV-1 EP721 proteins, p29, p40, and p48, were generated from the polyprotein sequences. The ORF-A polyprotein was divided into proteins p29 and p40 according to the previously identified cleavage site at Gly248/Gly249 (Jensen and Nuss, 2014). The ORF-B was split at Gly418/A419, creating p48 and RNA-dependent RNA polymerase (RdRp)-helicase. The models of the CHV-1 EP721 proteins were generated by the web-based service AlphaFold2 (Jumper et al., 2021) (https://colab.research.google.com/github/sokrypton/ColabFold/blob/main/AlphaFold2.ipynb) or Robetta (Baek et al., 2021) (https://robetta.bakerlab.org/) using the default settings. Models were visualized using Chimera (Pettersen et al., 2004) and ChimeraX (Pettersen et al., 2021). The secondary structure was extracted from the computed structure models using STRIDE (Frishman and Argos, 1995) or predicted by using PSRSM (Ma et al., 2018). Known catalytic, cleavage site, and mutation residues found in this study were mapped onto the secondary structure elements and/or the 3D computed models. In Chimera, the interaction between the residues was examined by the Find Clashes/Contacts tools. The Ser208 of the reference model was mutated to threonine using Rotamers tools and the Dunbrack 2010 rotamer library (Shapovalov and Dunbrack, 2011). The rotamer with the highest probability was selected. The energy-minimization was performed via Minimize Structure with unselected atoms fixed and the default setting. The standard residue charge model, AMBER ff14SB, was applied (Maier et al., 2015).

## 3 Results

### 3.1 Determination of hypovirulent isolates of *Cryphonectria parasitica* in South Tyrol

**Figure 1:**
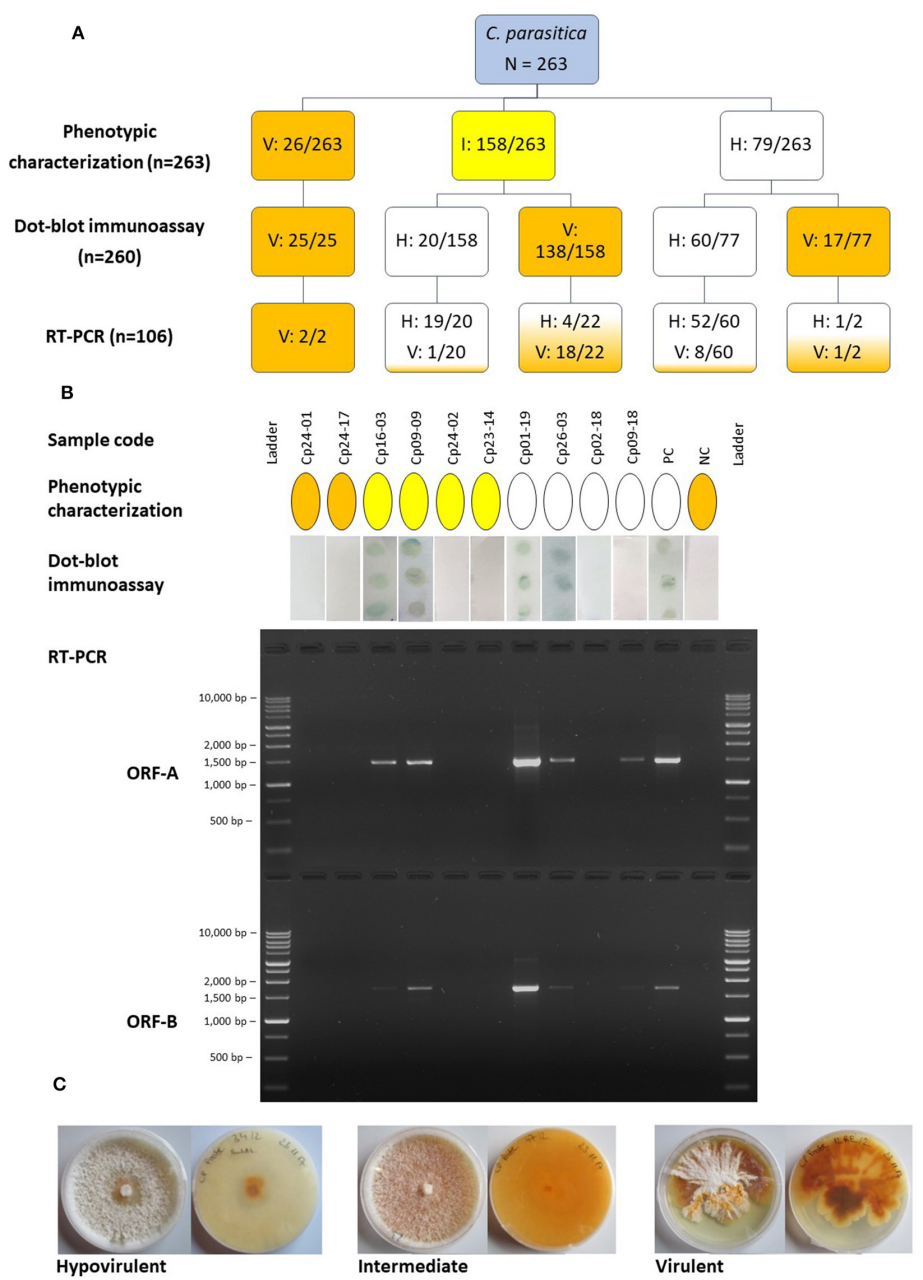
**(A)** Diagram representing the number of isolates of *Cryphonectria parasitica* designated as hypovirulent (H), virulent (V) or intermediate (I) using different detection approaches. The boxes are colored according to the category; blue, initially uncategorized isolates; white, hypovirulent; yellow, intermediate; orange, virulent. Numbers separated by a slash indicate the number of isolates assigned to a certain category compared to the number of isolates tested. **(B)** Comparison of the outcome of different detection approaches, phenotypic characterization, dot-blot immunoassay and RT-PCR of two different genomic regions, ORF-A and ORF-B, for ten representative samples. The same color codes as mentioned above were used to denote the phenotypic category of isolates as hypovirulent, intermediate or hypovirus negative/virulent. The presence of dots in the dot-blot immunoassay indicates the presence of dsRNA, whereas the samples with white membranes resulted negative. The PCR products were separated on a 1.5% agarose gel and the BenchTop 1 kb DNA Ladder (Promega, Madison, USA) was employed. **(C)** Upper and reverse views of representative cultures of *C. parasitica* on PDA depicting the hypovirulent, intermediate and virulent phenotype. PC, positive control (known hypovirulent isolate); NC, negative control (known virulent isolate).

Among 263 isolates of *C. parasitica* sampled throughout South Tyrol, 79 isolates were referred to as hypovirulent based on mycelial pigmentation, 26 were virulent and 158 were intermediates (Figure 1). Of the 263 isolates, 260 could be tested with the dot-blot immunoassay to assess the presence/absence of dsRNA. Three isolates (one virulent and two hypovirulent) were lost due to contamination and were not included in further analyses.

Results from the immunological assay demonstrated that the morphological approach was not always accurate; from the 79 isolates that were classified as hypovirulent with the phenotypic assay, only 60 were found to carry the dsRNA. However, isolates assigned as virulent based on their morphology always tested negative with the immunological test. The immunological test also helped to distinguish between a largely uncharacterized group of intermediate isolates that could be assigned neither to the virulent nor to the hypovirulent group based on phenotypic characteristics. Intermediate isolates usually resulted in the absence of dsRNA in the immunoassay; however, 12.6% of isolates showed the presence of dsRNA. Isolates of *C. parasitica* from all the chestnut growing regions in South Tyrol were found to have detectable dsRNA using the immunoassay. The presence of dsRNA, detected with the immunoassay, was validated through RT-PCR and gel electrophoresis. Clear bands of approximately 1.5 kb (ORF-A) and 1.7 kb (ORF-B) were observed following agarose gel electrophoresis (Figure 1). A total of 71 isolates were successfully amplified at least for one region (either ORF-A or ORF-B fragments) of CHV-1, which means that nine out of 80 isolates, which tested positive in the dot-blot immunoassay, have to be considered as false positives (Supplementary Table 3). Five out of 26 randomly selected isolates that were determined as hypovirus negative with the immunological assay resulted positive for the presence of CHV-1 in the RT-PCR analysis; consequently, 19.2% of the hypovirus negative isolates in the dot-blot immunoassay can be considered as false negatives.

Among 76 isolates that were CHV-1 positive in the RT-PCR assay, 67 were amplified for both ORFs, whereas nine were amplified for only one ORF. Related to the number of 260 isolates, the incidence of CHV-1 in South Tyrol was 29.2%, whereas 70.8% of isolates were CHV-1 negative. The occurrence of CHV-1 in different districts of South Tyrol ranged from 17.2% in Monticolo (5 of 29) to 41.9% in Oltradige-Bassa Atesina (13 of 31) (Table 1). A high variability was also found between chestnut stands and the incidence of CHV-1 ranged from 0 to 71.4%, excluding the stands in which the number of isolates was less than five (Supplementary Figure 2).

**Table 1:** Occurrence of *Cryphonectria hypovirus* 1 in different districts of South Tyrol.

**District in South Tyrol**	***N* isolates**	***N* of CHV-1**	**Frequency CHV-1 (%)**
Vinschgau—Val Venosta	59	16	27.1
Burggrafenamt—Burgraviato	53	14	26.4
Eisacktal—Valle Isarco	38	10	26.3
Salten-Schlern—Salto-Sciliar	50	18	36.0
Überetsch-Unterland—Oltradige-Bassa Atesina	31	13	41.9
Montiggler Wald—Monticolo	29	5	17.2
South Tyrol	260	76	29.2

### 3.2 Sequencing of nucleic acid fragments of CHV-1

Of all the amplicons obtained with ORF-A and ORF-B primers, cDNA sequences could be obtained for at least one ORF. The exception was one isolate, where sequencing failed for both regions. Sixty-nine and 65 sequences were obtained for ORF-A and ORF-B fragments, respectively, whereas 63 were sequenced for both ORFs. Sequences from both forward and reverse primers were assembled. The identities of all sequences from South Tyrol were confirmed with a BLAST analysis against the GenBank database (https://www.ncbi.nlm.nih.gov). BLAST results for all sequences showed more than 99% of sequence identity to different CHV-1 entries. However, no sequence from South Tyrol was identical to previously sequenced isolates of CHV-1 and all haplotypes were unique to the region. While all the sequences could be confirmed as CHV-1, 22 sequences of ORF-A and nine sequences of ORF-B were removed from phylogenetic analysis because of shorter sequence lengths or missing nucleotide data.

### 3.3 Nucleic acid sequence variation of CHV-1 in South Tyrol

Forty-seven partial CHV-1 sequences with a length of 1,202 bp were obtained for the genomic region ORF-A. A total of 23 different haplotypes were found among 47 sequences of CHV-1, with a haplotype diversity of 0.9270 ± 0.0230. No indels were found among the sequences analyzed and substitutions were the only type of mutation observed. One hundred four nucleotide positions were polymorphic, whereas 1,098 positions were monomorphic. Nucleotide diversity per site (*Pi*) was 0.0115 ± 0.0005 and the average number of nucleotide differences per pair of sequences within all the haplotypes (*k*) was 13.8. The haplotypes differed from each other in 1–27 nucleotides. Fifty-six partial CHV-1 sequences with a length of 1,194 bp were obtained for the genomic region ORF-B. A total of 30 different haplotypes were found among 56 sequences of CHV-1 with a haplotype diversity of 0.9610 ± 0.0001. Again, no indels were present and substitutions were the only type of mutation observed in the analyzed sequences. Eighty nucleotide positions were polymorphic, whereas 1,109 positions were monomorphic. In addition, five positions had missing data so they were neither considered monomorphic nor polymorphic. The observed *Pi* was 0.0070 ± 0.0001 and the average number of nucleotide differences per pair of sequences within all the haplotypes (*k*) was 8.4. The haplotypes differed from each other in 1–28 nucleotides.

A high degree of variation in the occurrence of haplotypes was observed; for example, HA1 was found ten times, whereas 15 different haplotypes based on ORF-A fragment were found just once (Supplementary Table 4). HB17 was found in seven different isolates and 19 different haplotypes based on ORF-B fragment were found just once (Supplementary Table 5). After combining the haplotype information of 40 sequences of partial ORF-A (1,202 bp) and ORF-B (1,194 bp) fragments, 24 different haplotypes were found (Supplementary Table 6). 21.3% of ORF-A sequences, 12.5% of ORF-B sequences and 15% of combined ORF-A and ORF-B sequences belonged to single haplotypes, HA1, HB17, HAB3, respectively, which were most dominant in South Tyrol (Table 2). No evidence of recombination among the partial sequences of ORF-A and ORF-B of CHV-1 subtype I was found when analyzing the data with the software RDP4, which demonstrated that recombination did not play a major role in the evolution of CHV-1 subtype I and mutations were the main source of diversity.

**Table 2:** Number of haplotypes of CHV-1 found for open reading frames ORF-A, ORF-B and combined sequences of ORF-A and ORF-B.

**Genomic region**	**N sequences**	**N haplotypes**	**Most dominant haplotype (%)**	**HD^a^**	**Pi^b^**	**k^c^**
ORF-A	47	23	HA1 (21.3)	0.9270	0.0115	13.8
ORF-B	56	30	HB17 (12.5)	0.9610	0.0070	8.4
ORF-A and ORF-B	40	24	HAB3 (15.0)	n.a.	n.a.	n.a.

a Haplotype diversity among all sequences.

b Nucleotide diversity per site.

c Average number of nucleotide differences per pair of sequences within all the haplotypes.

n.a., not applicable.

### 3.4 Phylogenetic relationships of CHV-1 from South Tyrol

All partial ORF-A and ORF-B sequences of CHV-1 obtained in this study clustered together with reference sequences of CHV-1 subtype I. The Italian cluster was clearly separated from other CHV-1 subtypes such as F1, F2 and D, whereas the Chinese subtype was the most distantly related (Supplementary Figure 3).

**Figure 2:**
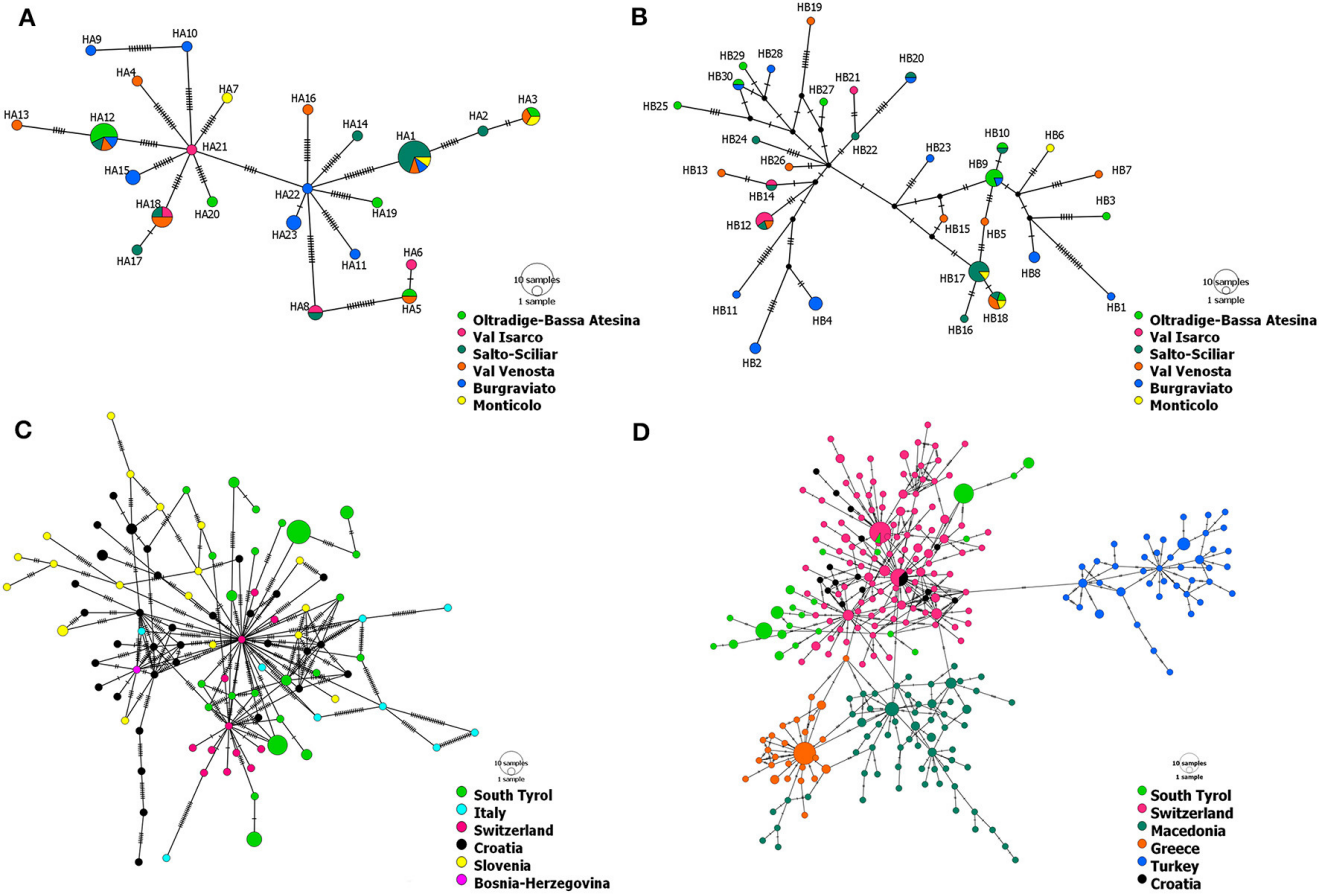
Haplotype networks of CHV-1 present in South Tyrol based on 1,202 bp of ORF-A **(A)** and 1,194 bp of ORF-B **(B)**. Haplotype networks showing the relationship of CHV-1 subtype I from South Tyrol and from different European countries were based on partial sequences of ORF-A and comprise 131 sequences with a length of 1,160 bp **(C)** and 379 sequences with a length of 561 bp **(D)**. Haplotype networks were drawn in POPART (Bandelt et al., 1999). The diameters of the circles represent the number of isolates or sequences found per haplotype and the color of circles represents the district, region or country of sampling, whereas black dots represent theoretical median vectors added by the software. Each perpendicular line on the branches indicates a mutational step.

Haplotype networks based on ORF-A and ORF-B containing only sequences of CHV-1 from South Tyrol pointed to the presence of two clusters (Figures 2A, B). No particular geographic pattern was observed in South Tyrol, neither for the clusters nor for the distribution of haplotypes.

Haplotype networks exclusively containing sequences of subtype I were constructed to better represent the relationship within the Italian subtype. The minimum spanning networks of CHV-1 subtype I based on ORF-A sequences from different European countries supported the presence of two clusters in South Tyrolean CHV-1. The same isolates were grouped in each of two clusters as observed in the haplotype network of South Tyrol (Figure 2). Haplotypes from South Tyrol were closely related to the Swiss haplotypes, indicating a potential relationship between populations. Haplotypes belonging to South Tyrol, Italy, Switzerland, Croatia and Bosnia clustered together, as shown in Figure 2C. Four clusters were observed, Swiss, Croatian and South Tyrolean haplotypes clustered together, whereas Turkish, Greek and Macedonian haplotypes represented each cluster separately (Figure 2D). As the length of ORF-A sequences was reduced from 1,160 bp (Figure 2C) to 561 bp (Figure 2D), the number of haplotypes found in South Tyrol and used in both analyses did not change (23). One haplotype (HA16) found in the region showed sequence identity with a haplotype from Switzerland (Figure 2D). This haplotype was detected in a single isolate found in Val Venosta, South Tyrol.

### 3.5 Molecular and evolutionary interpretation of substitutions found in the open reading frames of CHV-1

**Figure 3:**
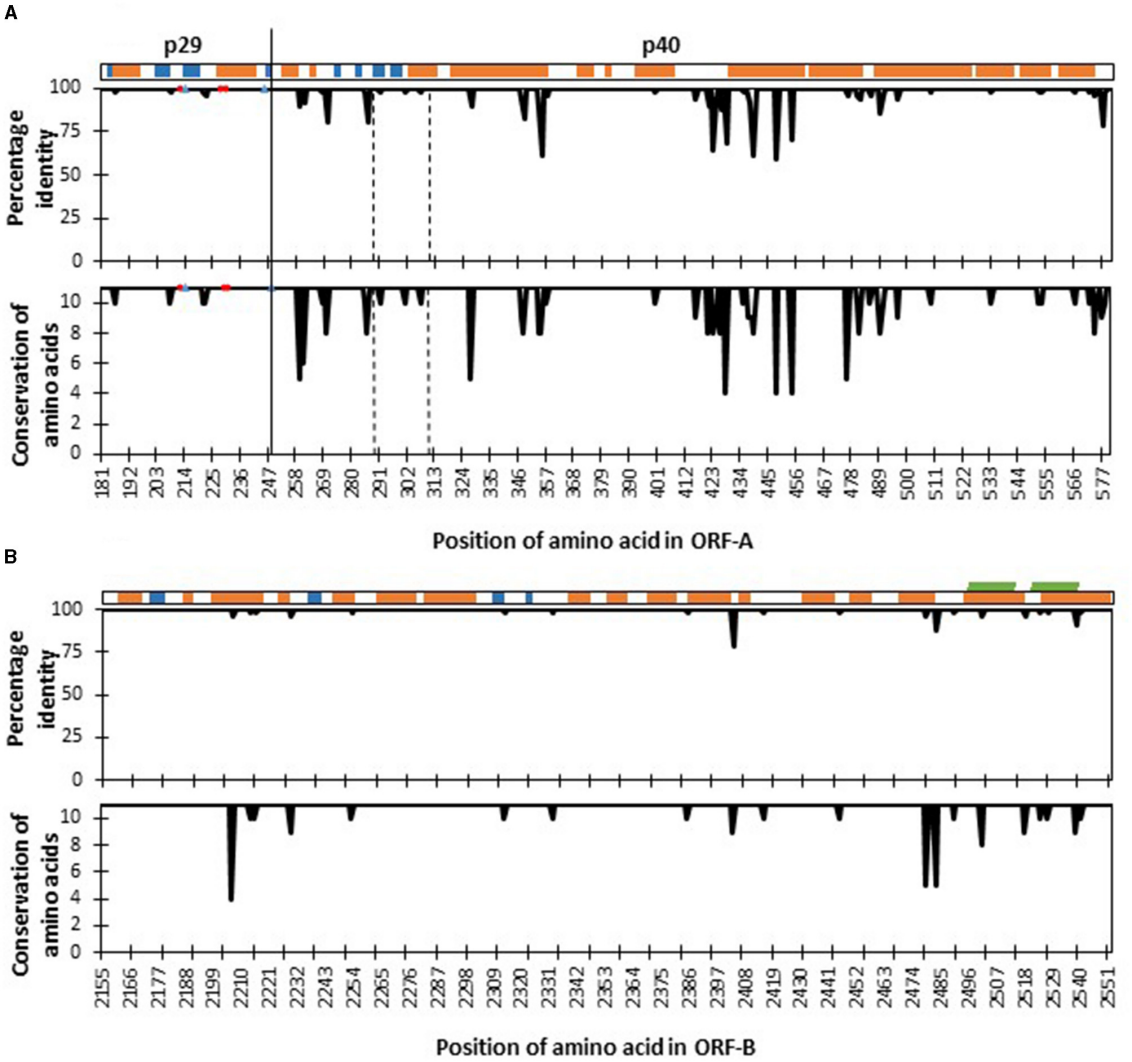
Four hundred amino acid positions [from amino acid 181 (tryptophan) to amino acid 580 (asparagine)] of the fragment of polyprotein ORF-A **(A)** and 398 amino acids [from amino acid 2,155 (leucine) to amino acid 2,552 (Glycine)] of polyprotein ORF-B **(B)** of *Cryphonectria hypovirus* 1 as compared to reference genome EP721 (DQ861913). The secondary structure elements of the polyprotein are shown at the top: α-helices (orange), β-strands (blue), and transmembrane (green). Percentage identity agreement among sequences of CHV-1 found in South Tyrol are represented in the upper plots, while amino acid conservation levels based on their physico-chemical properties are shown in the lower plots, which ranges from 0 to 11, and the latter value is the highest degree of conservation. Red circles in the ORF-A plots represent amino acid sites that are important for autocatalytic cleavage, whereas blue triangles show sites that affect the strength of autocatalysis. The functional domain of p40 protein from Thr^288^ to Arg^312^ is located between the two dashed vertical lines. The solid vertical line denotes the cleavage site separating proteins p29 and p40.

Mutations of two kinds were found in the nucleotide sequences of CHV-1 from South Tyrol; synonymous substitutions, which did not alter the amino acid and non-synonymous substitutions, which altered the amino acid. Translation of partial ORF-A sequences of CHV-1 from South Tyrol resulted in a polypeptide fragment of 400 amino acids in which 48 synonymous and 51 non-synonymous polymorphic sites were observed. Translation of partial ORF-B sequences produced a polypeptide fragment of 398 amino acids with 81 polymorphic sites. Of these, 61 sites represented synonymous mutations, whereas only 20 non-synonymous mutations were found in ORF-B. Hence, amino acid conservation was higher in ORF-B than in ORF-A (Figure 3).

**Figure 4:**
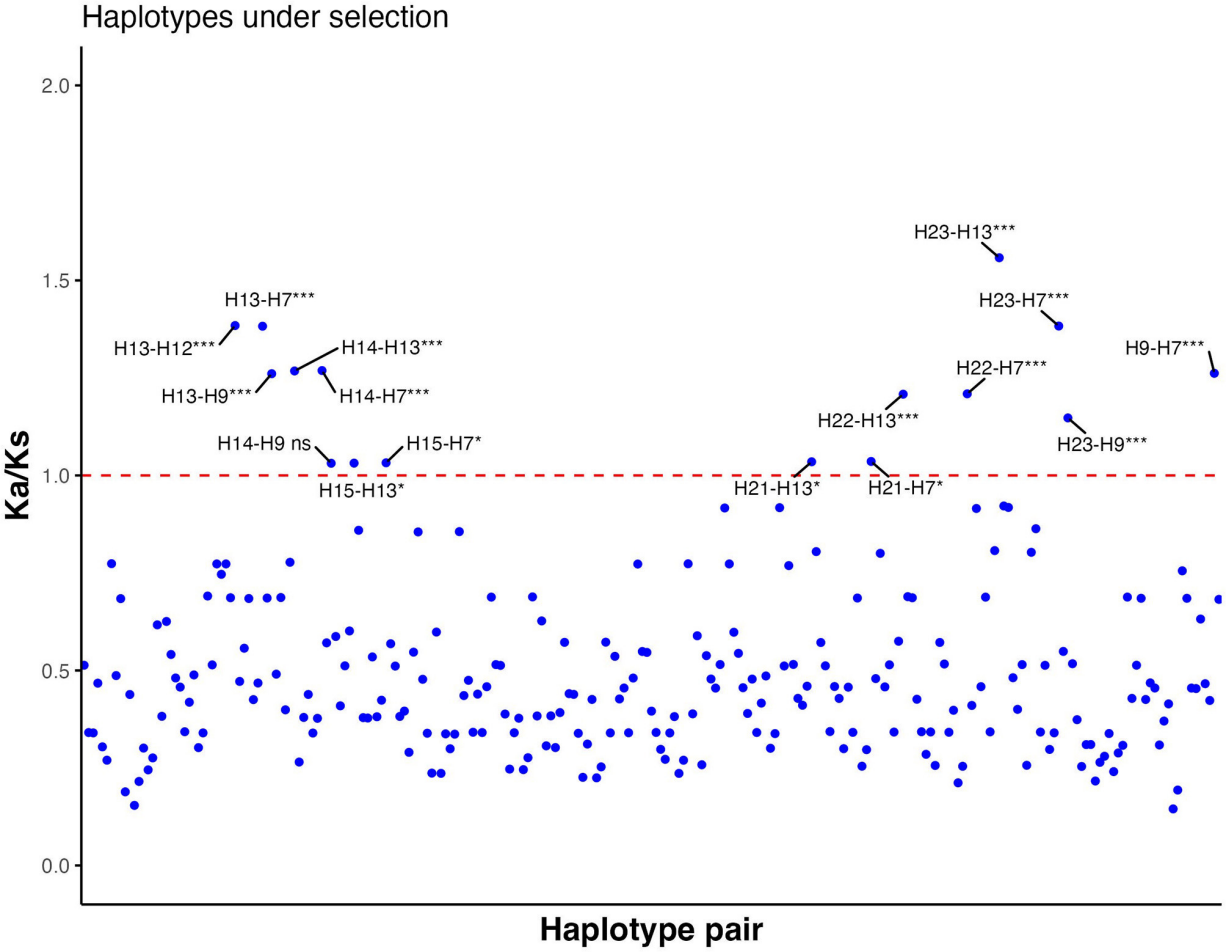
Ratio between non-synonymous substitution rate (K_a_) and synonymous substitution rate (K_s_) of all possible haplotype pair combinations based on partial sequences of ORF-A. Values significantly higher than 1 indicate positive selection, whereas values significantly lower than 1 show negative selection. If K_a_/K_s_ is not significantly different from 1, neutral evolution is expected. Each blue dot is a data point of each K_a_/K_s_ value. The red line shows the threshold value (K_a_/K_s_ = 1). ns, not significant; *, significant (*p*-value < 0.05); ***, highly significant (*p*-value < 0.001).

Each haplotype was compared with all other haplotypes to calculate pairwise K_a_/K_s_ ratios. The majority of combinations of ORF-A haplotypes had K_a_/K_s_ ratios lower than 1 (Figure 4; Supplementary Table 7). However, 15 combinations demonstrated positive selection (K_a_/K_s_ values significantly higher than 1); for example, HA12, found in seven isolates, had a K_a_/K_s_ ratio of 1.38 when paired with HA13 (Supplementary Table 8). Other haplotypes that were probable to be under positive selection (K_a_/K_s_ significantly higher than 1) based on ORF-A fragment were HA7, HA9, HA13, HA14, HA15, HA21, HA22, and HA23. In the ORF-B fragment, all the combinations between isolates showed K_a_/K_s_ lower than 1 and no strains appeared to be under positive selection (data not shown). A major part of the mutations observed for ORF-B fragment was also found to be synonymous. Within the haplotypes of CHV-1 based on ORF-A sequences found in South Tyrol, 21 amino acid changes were intolerant and predicted that these amino acid changes could affect the phenotype (Supplementary Table 9). All of these intolerant positions were found in rare haplotypes that were found just once. However, some isolates had more than one intolerant substitution. The following haplotypes had intolerant substitutions in ORF-A fragment: HA4, HA7, HA9, HA10, HA11, HA13, HA14, and HA20. Based on a combined approach of SIFT and K_a_/K_s_, haplotypes HA7, HA9, HA13 and HA14 were identified as VOIs as they were common in both analyses. Although HA12 did not have an intolerant mutation, it was also identified as VOI because of a significantly higher K_a_/K_s_ value than 1, a high number of isolates carrying this ORF-A haplotype and a high sequence identity with another VOI (HA13).

To identify the proper functioning of proteins, a comparison of known functional amino acid sites involved in the autocatalytic activity of polyproteins was performed. All haplotypes were highly conserved in amino acid sites that were important for the autocatalytic cleavage, such as His^215^ and Gly^248^ and amino acid sites that affect the strength of autocatalysis such as His^212^, Leu^230^ and Gln^233^. However, the functional domain of p40 from Thr^288^ to Arg^312^ was not 100% conserved and three non-synonymous mutations were found (Figure 3). Specifically, CHV-1 haplotype HA4 displayed variability at position 291, where amino acid serine was substituted with phenylalanine (Supplementary Table 9). As the Grantham score for this substitution was 155, it was designated as a radical change. Haplotype HA7 had the amino acid asparagine instead of serine at position 301 with Grantham's distance of 46 and was therefore ranked as conservative. HA11 had the amino acid valine instead of methionine at position 307 and was also designated as conservative (Grantham score of 21).

The p29 and p40 proteins are known to play a critical role in causing the hypovirulence to the host. Therefore, we used modeling to predict the structure of both proteins and to shed light on how mutations could influence the protein structure. The p29 AlphaFold model is a helix-rich structure with 13 helices and 4 β-sheets (2 small β-sheets). It has a range of the predicted local distance difference test (pLDDT), or confidence score, from low to high (Supplementary Figure 4). The area of interest, including the catalytic residues and a cleavage site was predicted with a high confidence score, allowing a possible conformation of the active site to be observed. The cleavage site Gly248 is flanked by the catalytic residues Cys162 and His215. Even though these residues are conserved, Ser208Thr, a neighboring substitution mutation, was found to be close to His215. The Ser208 was manually mutated to threonine, and the highest probability rotamer gave a similar conformation to the wild-type serine contacting the H215. However, following energy minimization, at the local energy minimum, the side chain of histidine 215 changed its conformation and the clash was relieved, indicating that the contact may not take place in the structure. It is nonetheless important to note that the substitution may have an impact on the autocleavage of the ORF-A and that the hypothesis should be further experimentally validated.

For p40, Alphafold provided a poor overall confidence score of 50–60. As a result, the Robetta model was utilized instead, which has a low error score in the functional domain (Thr288-Arg312). It should be mentioned that both AlphaFold and Robetta predict a similar conformation for this area with template modeling score (TM-score) of 0.51. The p40 is also helix-rich, with 15 helices and 4 β-sheets. Mutations are only mapped to the functional domain, and they are found throughout the helix, β-sheet, and unspecified regions. However, there is no interaction between these wild-type residues. Without the molecular details of how the protein functions, it is unclear how these mutations could affect the protein. For the ORF-B, sequencing fragments (398 amino acids) were mapped onto the region between the RdRp and the helicase domain. The fragment contains the C-terminal part, 40 amino acids of the RdRp and the undefined region. There is no mutation observed in the RdRp domain (Figure 3).

## 4 Discussion

Phenotypic screening of *C. parasitica* isolates for the presence of CHV-1, although widely used, often leads to high error rates because many viral infections do not induce phenotypic changes in the host (Ghabrial, 1998). In the study of Castaño et al. (2014), for example, among 179 hypovirulent strains of *C. parasitica* identified by phenotypic characterization, the presence of dsRNA was found in only 35 isolates. This study showed that the pigmentation is not a reliable approach to detect hypovirulence. In our study also, phenotypic data of *C. parasitica* cultures might have given very different results as compared to the immunoassay because only 26 isolates were clearly identified as virulent based on morphology. Using the implemented immunoassay, however, we were able to screen a large number of *C. parasitica* isolates for the presence/absence of mycovirus, independent of the colony phenotype. Verified by RT-PCR, the immunoassay could reasonably well-detect the presence of dsRNA of CHV-1 infecting *C. parasitica*. Although Peever et al. (1998) also used an immunological method to detect the presence of dsRNA in *C. parasitica*, their method included nucleic acid extraction, purification and, separation of dsRNA through electrophoresis before applying the nucleic acid on the nitrocellulose membrane. In the protocol described by Yaegashi et al. (2011) and adapted for the analysis of *C. parasitica*, these labor and time-consuming steps could be skipped by directly applying a crude extract of fungal mycelium to the nitrocellulose membrane. However, nine isolates were false positives, which could be due to the presence of other RNA viruses in *C. parasitica*, as many other species of RNA viruses can infect the fungus (Suzuki et al., 2018; Forgia et al., 2021). As we used primers specific for CHV-1, the presence of other members of the former genus *Hypovirus* or mycoviruses from other families (*Totiviridae, Partitiviridae, Narnaviridae* etc.) might have given a positive result in the immunoassay test but a negative one in the RT-PCR test. To confirm this hypothesis, such isolates should be further investigated by next-generation sequencing for virus detection.

False negatives in our study might be explained due to the low signal produced by the assay, as a high concentration of the viral dsRNA is needed to visually detect the antigen-antibody reaction on a membrane. Nevertheless, the dot-blot immunoassay saved significant time and resources compared to the previous immunological methods or RT-PCR system. This protocol could be further developed into an ELISA assay or a lateral flow test for the rapid detection of mycoviruses for large-scale screening in the laboratory or even applied in the field.

Biocontrol of chestnut blight through natural or artificially introduced hypovirulence has been rather successful in Europe (Heiniger and Rigling, 1994) and it is common to find CHV-1 occurrence of more than 50% in the fungal population (Krstin et al., 2008, 2011; Robin et al., 2010; Bryner and Rigling, 2012; Rigling and Prospero, 2018). A low prevalence of hypovirulent isolates in South Tyrol (29.2% on average) as compared to other European regions (for example, central Italy with 38.4% of hypovirulence) represents a potential threat to local chestnut trees (Murolo et al., 2018). As CHV-1 is preferentially transmitted between the same VC type, a high VC-type diversity of the fungal host in the region (Ahmad and Baric, 2022a) might be the reason of low CHV-1 occurrence. Sexual spores (ascospores) of *C. parasitica* are normally free from hypoviruses (Prospero et al., 2006), therefore, frequent sexual reproduction in the region might be another possible explanation of this low occurrence as evidenced by a mating-type ratio close to 1:1 (Ahmad and Baric, 2022a) and high sexual reproduction inferred through microsatellite analysis (Ahmad and Baric, 2022b). The low level of hypovirulence occurrence in South Tyrol could also be influenced by the current control strategies of chestnut blight that rely exclusively on sanitation and pruning of infected chestnut trees. In contrast to South Tyrol, many other European regions successfully use the artificial application of hypovirulent fungal strains as biocontrol agents against chestnut blight (Heiniger and Rigling, 1994, 2009; Robin et al., 2000; Prospero and Rigling, 2016; Coelho et al., 2021). Although artificial inoculation of hypovirulent strains of *C. parasitica* was performed in South Tyrol in the 1990s (Maresi et al., 1993), more than 25 years have passed since the last application. As a loss of hypovirulence over time was observed in other regions (Bryner et al., 2014), this could also have happened in South Tyrol. In contrast, a recent study showed that virus infection rates were stable over time in the populations of Switzerland (Stauber et al., 2022). A previous study performed in the 1990s also found that the occurrence of hypovirulence (indicated by healing cankers) in South Tyrol was the lowest compared to other Italian populations and explained that it might be due to the isolation of chestnut stands in the region (Heiniger and Rigling, 1994). Based on our data and previous studies, we, therefore, recommend implementing biocontrol measures in the region based on hypovirulence against *C. parasitica* in order to preserve local chestnut trees, especially in those chestnut stands and areas where hypovirulent strains were absent or occurred at a very low frequency.

A high degree of genetic variability of CHV-1 in the region was found based on the sequencing of ORF-A and ORF-B fragments. Similar findings were observed in other well-established European populations of *C. parasitica* (Liu et al., 2007; Bryner et al., 2012; Feau et al., 2014; Krstin et al., 2020; Ježić et al., 2021). One reason for this high diversity is that RNA viruses have particularly high mutation rates, almost a million times higher than their hosts (Duffy, 2018). Therefore, a high degree of genetic diversity of CHV-1 appears to be a natural progression in established European populations. In contrast, a low diversity was observed in newly established populations, such as in Great Britain (Romon-Ochoa et al., 2022).

All the identified isolates of CHV-1 found in South Tyrol clustered together with reference isolates of subtype I and we did not find any other subtype of CHV-1. This is not surprising, first, because of the geographical location of South Tyrol, which is in close proximity with other north Italian populations and second, because subtype I is the most widespread CHV-1 in Europe. A high prevalence of subtype I is thought to be because of its greater ecological fitness, resulting in better dissemination and higher potential to establish in different populations than other subtypes (Robin et al., 2010). Within subtype I, phylogeographic studies indicated a close relationship of South Tyrolean isolates with neighboring regions of Switzerland, Northern Italy and other European countries where subtype I is usually found (Krstin et al., 2020). The haplotypes found in this study were similar to many Swiss haplotypes, further demonstrating the close relationship of the populations from both regions. As previously observed for its fungal host, *C. parasitica*, that multiple introductions might have happened to South Tyrol, CHV-1 sequencing concurred with the previous studies and suggested that human-mediated introduction may have played a role in increasing the variability of CHV-1 (Ahmad and Baric, 2022a,b).

The genome of CHV-1 consists of two ORFs (ORF-A and ORF-B) encoding for polyproteins that are auto-catalytically cleaved (Nuss, 2005). The cleavage of ORF-A polyprotein p69 results in two proteins, p29 and p40, which impact the decrease of fungal pigmentation and sporulation, whereas p29 also decreases laccase production and increases the viral transmission to asexual spores (Suzuki and Nuss, 2002; Suzuki et al., 2003). Most nucleotide substitutions are neutral and have little or no effect on the proper functioning of the protein, but some non-synonymous mutations can have severe consequences (Yates and Sternberg, 2013). We found non-synonymous and intolerant substitutions in amino acids coding for both polyproteins showing that these substitutions might substantially change their functioning. ORF-A fragment displayed a higher amino acid mutation rate than ORF-B as well as higher values of K_a_/K_s_. The conservation of ORF-B fragment might be related to its crucial role in viral replication i.e., by producing RNA-dependent RNA polymerase. Within ORF-A polyprotein, sites that are important for its autocatalytic cleavage were found to be highly conserved in our study, a result that is in line with a previous study (Krstin et al., 2020). Conversely, the p40 functional domain was not conserved in the CHV-1 sequences identified in South Tyrol and one radical and two non-radical amino acid substitutions were detected, leading to the hypothesis that these haplotypes might have a low functional p40 protein. The radical substitution was only found in a single sequence, which is consistent with a major impact on the stability of the protein. Substitutions in the functional domain of p40 were previously found among different CHV-1 subtypes; however, within subtype I, it remained largely conservative (Krstin et al., 2020). Predicted protein structures also showed that p29 and p40 might have some mutations that would affect the polyprotein and its cleavage. The detailed function and mode of action of p40 protein remain unclear, making it harder to interpret how the mutant strain would change its host's phenotype. Nevertheless, based on the known role of p40 in hypovirulence (Suzuki and Nuss, 2002), we suggest that some mutations in ORF-A polyprotein might be under negative selection. The fact that each of these mutations was found just in single isolates further strengthens this hypothesis.

The dominance of a few haplotypes in the region was observed; for example, three haplotypes based on ORF-A fragment accounted for almost 45% of total CHV-1 sequences obtained from South Tyrol. There are several hypotheses to address why some strains of hypoviruses are rare, whereas others are widespread. A reason at the base of this finding might be that the dominant haplotypes infect the more dominant VC-type strains of the fungus, giving them an ecological advantage over haplotypes present in non-dominant VC types. The haplotypes present in the dominant VC types are more likely to find another host than rare ones because the virus is preferentially transmitted through a hyphal anastomosis between VC-type compatible strains (Choi et al., 2012). However, when the dominant haplotypes, HA1 and HA12, were checked for the VC type of their fungal hosts (Ahmad and Baric, 2022a), no such correlation was found (data not shown). Another hypothesis to explain the dominance of some haplotypes in the region is that the amino acid changes caused by mutations in the open reading frames might have given a selective advantage to particular haplotypes to better establish themselves compared to others. If a mutation is independently multiplying several times, it increases the weight of evidence that the mutation is beneficial for the virus (Kupferschmidt, 2021). Interestingly, one VOI (HA12) was found in almost 15% of total isolates, further showing that this haplotype might have attained a better functioning of the ORF-A polyprotein. A significantly higher K_a_/K_s_ value found in different haplotype combinations (including HA12) also demonstrated that some haplotypes are under positive selection and might have better ecological fitness. Changes in the phenotype of the fungus induced by CHV-1 subtype I due to substitutions were already observed in Croatia (Krstin et al., 2020). In other positive-sense single-strand RNA viruses, like the Chikungunya virus and SARS-CoV-2, some mutations have helped the viruses to better disseminate and rapidly establish themselves (Tsetsarkin et al., 2007; Kupferschmidt, 2021). Even though HA1 neither displayed any intolerant mutation nor a significantly high K_a_/K_s_ value, it was the most dominant haplotype, demonstrating the fitness of the virus of this haplotype as a possible effective biological control agent. Therefore, HA1 should also be regarded as VOI. Positive selection among CHV-1 subtype I was already detected, suggesting that host adaptation might have occurred in other populations (Feau et al., 2014). However, most of the VOIs were only found once, making a significant selection advantage less likely, as a high K_a_/K_s_ ratio does not guarantee a positive selection (Roth and Liberles, 2006), hence resulting in less suitable strains for biocontrol purposes. HA4, HA7, and HA9 fall into this category due to a possible compromised ORF-A polyprotein functioning. We recommend that all identified VOIs be investigated further for their role in the functioning of the ORF-A polyprotein and their impact on the fitness of the fungal host.

## 5 Conclusions

The present study pointed out a low occurrence of hypovirulence in South Tyrol, indicating a need to implement effective biocontrol strategies through the artificial application of hypovirulent *C. parasitica* strains in the chestnut stands of the region. In addition, some haplotypes were identified as VOIs because of the presence of radical and intolerant mutations. Some identified non-synonymous mutations might have a selection advantage over others. Based on these results, two haplotypes, HA1 and HA12, should be specifically investigated in a future study for their biocontrol properties. It must be considered that the present study was based on sequence analysis and did not prove that the reported substitutions change the protein functioning causing substantial changes in the host phenotype. Therefore, further *in-vitro* and *in-vivo* studies are recommended to better understand the effect of these mutations on the fungal phenotype and the functioning of proteins.

## Data availability statement

The datasets presented in this study can be found in online repositories. The names of the repository/repositories and accession number(s) can be found in the article/Supplementary material.

## Author contributions

FA: Conceptualization, Formal analysis, Investigation, Methodology, Validation, Visualization, Writing—original draft. ST: Formal analysis, Methodology, Validation, Visualization, Writing—review & editing. TP: Formal analysis, Investigation, Visualization, Writing—review & editing. SB: Conceptualization, Data curation, Formal analysis, Funding acquisition, Investigation, Methodology, Project administration, Supervision, Validation, Visualization, Writing—review & editing.

## References

[B1] Aguin O. Mata M. Mansilla J. P. Romero A. (2005). Occurrence and diversity of vegetative compatibility types of *Cryphonectria parasitica* in Galicia (NW Spain). Acta Hortic. 693, 597–604. 10.17660/ActaHortic.2005.693.7917682780

[B2] Ahmad F. Baric S. (2022a). Genetic diversity of *Cryphonectria parasitica* causing chestnut blight in South Tyrol (northern Italy). Eur. J. Plant Pathol. 162, 621–635. 10.1007/s10658-021-02425-2

[B3] Ahmad F. Baric S. (2022b). Microsatellite analysis revealing high genetic diversity of the chestnut blight fungus in South Tyrol (northern Italy). Forests 13:344. 10.3390/f13020344

[B4] Akilli S. Serçe Ç. Katirciouglu Y. Z. Maden S. Rigling D. (2013). Characterization of hypovirulent isolates of the chestnut blight fungus, *Cryphonectria parasitica* from the Marmara and Black Sea regions of Turkey. Eur. J. Plant Pathol. 135, 323–334. 10.1007/s10658-012-0089-z

[B5] Allemann C. Hoegger P. Heiniger U. Rigling D. (1999). Genetic variation of *Cryphonectria* hypoviruses (CHV1) in Europe, assessed using restriction fragment length polymorphism (RFLP) markers. Mol. Ecol. 8, 843–854. 10.1046/j.1365-294X.1999.00639.x 10368967

[B6] Anagnostakis S. (1987). Chestnut blight: the classical problem of an introduced pathogen. Mycologia 79:23. 10.1080/00275514.1987.12025367

[B7] Aramburu J. Moreno P. (1994). Detection of double-stranded RNA (DSRNA) in crude extracts of virus-infected plants by indirect ELISA. J. Phytopathol. 141, 375–385. 10.1111/j.1439-0434.1994.tb04512.x

[B8] Baek M. DiMaio F. Anishchenko I. Dauparas J. Ovchinnikov S. Lee G. R.. (2021). Accurate prediction of protein structures and interactions using a three-track neural network. Science 373, 871–876. 10.1126/science.abj8754 34282049 PMC7612213

[B9] Bandelt H. J. Forster P. Röhl A. (1999). Median-joining networks for inferring intraspecific phylogenies. Mol. Biol. Evol. 16, 37–48. 10.1093/oxfordjournals.molbev.a026036 10331250

[B10] Biraghi A. (1946). Il cancro del castagno causato da *Endothia parasitica*. Ital. Agric. 7, 1–9.

[B11] Bissegger M. Rigling D. Heiniger U. (1997). Population structure and disease development of *Cryphonectria parasitica* in European chestnut forests in the presence of natural hypovirulence. Phytopathology 87, 50–59. 10.1094/PHYTO.1997.87.1.50 18945153

[B12] Bryner S. F. Prospero S. Rigling D. (2014). Dynamics of *Cryphonectria hypovirus* infection in chestnut blight cankers. Phytopathology 104, 918–925. 10.1094/PHYTO-03-13-0069-R 24601984

[B13] Bryner S. F. Rigling D. (2012). Hypovirus virulence and vegetative incompatibility in populations of the chestnut blight fungus. Phytopathology 102, 1161–1167. 10.1094/PHYTO-01-12-0013-R 22857516

[B14] Bryner S. F. Rigling D. Brunner P. C. (2012). Invasion history and demographic pattern of *Cryphonectria hypovirus* 1 across European populations of the chestnut blight fungus. Ecol. Evol. 2, 3227–3241. 10.1002/ece3.429 23301186 PMC3539014

[B15] Castaño C. Bassie L. Oliach D. Gómez M. Medina V. Liu B.. (2014). *Cryphonectria hypovirus* 1 (CHV-1) survey reveals low occurrence and diversity of subtypes in NE Spain. For. Pathol. 45, 51–59. 10.1111/efp.12131

[B16] Chen C. Chen H. Zhang Y. Thomas H. R. Frank M. H. He Y.. (2020). TBtools: an integrative toolkit developed for interactive analyses of big biological data. Mol. Plant 13, 1194–1202. 10.1016/j.molp.2020.06.009 32585190

[B17] Chiba S. Velasco L. Ayllón M. A. Suzuki N. Lee-Marzano S.-Y. Sun L.. (2023). ICTV Virus taxonomy profile: hypoviridae 2023. J. Gen. Virol. 104:001848. 10.1099/jgv.0.001848 37192093 PMC12643068

[B18] Choi G. H. Dawe A. L. Churbanov A. Smith M. L. Milgroom M. G. Nuss D. L. (2012). Molecular characterization of vegetative incompatibility genes that restrict hypovirus transmission in the chestnut blight fungus *Cryphonectria parasitica*. Genetics 190, 113–127. 10.1534/genetics.111.133983 22021387 PMC3249360

[B19] Choi G. H. Nuss D. (1992). Hypovirulence of chestnut blight fungus conferred by an infectious viral cDNA. Science 257, 800–803. 10.1126/science.1496400 1496400

[B20] Coelho V. Nunes L. Gouveia E. (2021). Short and long term efficacy and prevalence of *Cryphonectria parasitica* hypovirulent strains released as biocontrol agents of chestnut blight. Eur. J. Plant Pathol. 159, 769–781. 10.1007/s10658-021-02200-3

[B21] Del Carratore R. Aghayeva D. N. Ali-zade V. M. Bartolini P. Della Rocca G. Emiliani G.. (2021). Detection of *Cryphonectria hypovirus* 1 in *Cryphonectria parasitica* isolates from Azerbaijan. For. Pathol. 51:e12718. 10.1111/efp.12718

[B22] Djelouah K. Frasheri D. Valentini F. Donghia A. M. Digiaro M. (2014). Direct tissue blot immunoassay for detection of *Xylella fastidiosa* in olive trees. Phytopathol. Mediterr. 53, 559–564. 10.14601/Phytopathol_Mediterr-14603

[B23] Duffy S. (2018). Why are RNA virus mutation rates so damn high? PLoS Biol. 16:e3000003. 10.1371/journal.pbio.3000003 30102691 PMC6107253

[B24] Feau N. Dutech C. Brusini J. Rigling D. Robin C. (2014). Multiple introductions and recombination in *Cryphonectria hypovirus* 1: perspective for a sustainable biological control of chestnut blight. Evol. Appl. 7, 580–596. 10.1111/eva.12157 24944571 PMC4055179

[B25] Fedrigotti V. B. Fischer C. (2015). Sustainable development options for the chestnut supply chain in South Tyrol, Italy. Agric. Agric. Sci. Proc. 5, 96–106. 10.1016/j.aaspro.2015.08.014

[B26] Forgia M. Isgandarli E. Aghayeva D. N. Huseynova I. Turina M. (2021). Virome characterization of *Cryphonectria parasitica* isolates from Azerbaijan unveiled a new mymonavirus and a putative new RNA virus unrelated to described viral sequences. Virology 553, 51–61. 10.1016/j.virol.2020.10.008 33221630

[B27] Frishman D. Argos P. (1995). Knowledge-based protein secondary structure assignment. Prot. Struct. Funct. Genet. 23, 566–579. 10.1002/prot.340230412 8749853

[B28] Ghabrial S. A. (1998). Origin, adaptation and evolutionary pathways of fungal viruses. Virus Genes 16, 119–131. 10.1023/A:1007966229595 9562896 PMC7089520

[B29] Gobbin D. Hoegger P. J. Heiniger U. Rigling D. (2003). Sequence variation and evolution of *Cryphonectria hypovirus* 1 (CHV-1) in Europe. Virus Res. 97, 39–46. 10.1016/S0168-1702(03)00220-X 14550586

[B30] Grantham R. (1974). Amino acid difference formula to help explain protein evolution. Science 185, 862–864. 10.1126/science.185.4154.862 4843792

[B31] Heiniger U. Rigling D. (1994). Biological control of chestnut blight in Europe. Annu. Rev. Phytopathol. 32, 581–599. 10.1146/annurev.py.32.090194.00305317771259

[B32] Heiniger U. Rigling D. (2009). Application of the *Cryphonectria hypovirus* (CHV-1) to control the chestnut blight, experience from Switzerland. Acta Hortic. 815, 233–246. 10.17660/ActaHortic.2009.815.31

[B33] Hillman B. I. Suzuki N. (2004). Viruses in the chestnut blight fungus. Adv. Virus Res. 63, 423–472. 10.1016/S0065-3527(04)63007-7 15530566

[B34] Jensen K. S. Nuss D. L. (2014). Mutagenesis of the catalytic and cleavage site residues of the hypovirus papain-like proteases p29 and p48 reveals alternative processing and contributions to optimal viral RNA accumulation. J. Virol. 88, 11946–11954. 10.1128/JVI.01489-14 25100848 PMC4178723

[B35] Ježić M. Schwarz J. M. Prospero S. Sotirovski K. Risteski M. Curković-Perica M.. (2021). Temporal and spatial genetic population structure of *Cryphonectria parasitica* and its associated hypovirus across an invasive range of chestnut blight in Europe. Phytopathology 111, 1327–1337. 10.1094/PHYTO-09-20-0405-R 33417482

[B36] Jumper J. Evans R. Pritzel A. Green T. Figurnov M. Ronneberger O.. (2021). Highly accurate protein structure prediction with AlphaFold. Nature 596, 583–589. 10.1038/s41586-021-03819-2 34265844 PMC8371605

[B37] Khalifa M. E. Pearson M. N. (2014). Characterisation of a novel hypovirus from *Sclerotinia sclerotiorum* potentially representing a new genus within the Hypoviridae. Virology 464, 441–449. 10.1016/j.virol.2014.07.005 25108682

[B38] Krstin L. Katanić Z. Repar J. Ježić M. Kobaš A. Ćurković-Perica M. (2020). Genetic diversity of *Cryphonectria hypovirus* 1, a biocontrol agent of chestnut blight, in Croatia and Slovenia. Microb. Ecol. 79, 148–163. 10.1007/s00248-019-01377-9 31053974

[B39] Krstin L. Novak-Agbaba S. Rigling D. Ćurković-Perica M. (2011). Diversity of vegetative compatibility types and mating types of *Cryphonectria parasitica* in Slovenia and occurrence of associated *Cryphonectria hypovirus* 1. Plant Pathol. 60, 752–761. 10.1111/j.1365-3059.2011.02438.x

[B40] Krstin L. Novak-Agbaba S. Rigling D. Krajačić M. Ćurković Perica M. (2008). Chestnut blight fungus in Croatia: diversity of vegetative compatibility types, mating types and genetic variability of associated *Cryphonectria hypovirus* 1. Plant Pathol. 57, 1086–1096. 10.1111/j.1365-3059.2008.01905.x

[B41] Kumar S. Stecher G. Li M. Knyaz C. Tamura K. (2018). MEGA X: molecular evolutionary genetics analysis across computing platforms. Mol. Biol. Evol. 35, 1547–1549. 10.1093/molbev/msy096 29722887 PMC5967553

[B42] Kupferschmidt K. (2021). Fast-spreading UK virus variant raises alarms. Science 371, 9–10. 10.1126/science.371.6524.9 33384355

[B43] Li H. Bian R. Liu Q. Yang L. Pang T. Salaipeth L.. (2019). Identification of a novel hypovirulence-inducing hypovirus from *Alternaria alternata*. Front. Microbiol. 10:1076. 10.3389/fmicb.2019.01076 31156589 PMC6530530

[B44] Li W. H. Wu C. I. Luo C. C. (1984). Nonrandomness of point mutation as reflected in nucleotide substitutions in pseudogenes and its evolutionary implications. J. Mol. Evol. 21, 58–71. 10.1007/BF02100628 6442359

[B45] Liu F. X. Ding P. Xu C. X. Wang K. R. (2007). Genetic diversity of *Cryphonectria hypovirus* 1 in China, Japan and Italy. J. Phytopathol. 155, 662–669. 10.1111/j.1439-0434.2007.01292.x

[B46] Ma Y. Liu Y. Cheng J. (2018). Protein secondary structure prediction based on data partition and semi-random subspace method. Sci. Rep. 8:9856. 10.1038/s41598-018-28084-8 29959372 PMC6026213

[B47] Maier J. A. Martinez C. Kasavajhala K. Wickstrom L. Hauser K. E. Simmerling C. (2015). ff14SB: Improving the accuracy of protein side chain and backbone parameters from ff99SB. J. Chem. Theory Comput. 11, 3696–3713. 10.1021/acs.jctc.5b00255 26574453 PMC4821407

[B48] Maresi G. Minerbi S. Sottovia A. Turchetti T. (1993). Der Kastanienrinderkrebs in Sudtirol. AFZ 3, 140–144.

[B49] Martin D. P. Murrell B. Golden M. Khoosal A. Muhire B. (2015). RDP4: detection and analysis of recombination patterns in virus genomes. Virus Evol. 1:vev003. 10.1093/ve/vev003 27774277 PMC5014473

[B50] Milgroom M. G. Cortesi P. (2004). Biological control of chestnut blight with hypovirulence: a critical analysis. Annu. Rev. Phytopathol. 42, 311–338. 10.1146/annurev.phyto.42.040803.140325 15283669

[B51] Morozov A. Y. Robin C. Franc A. (2007). A simple model for the dynamics of a host–parasite–hyperparasite interaction. J. Theor. Biol. 249, 246–253. 10.1016/j.jtbi.2007.05.041 17884101

[B52] Murolo S. De Miccolis Angelini R. M. Faretra F. Romanazzi G. (2018). Phenotypic and molecular investigations on hypovirulent *Cryphonectria parasitica* in Italy. Plant Dis. 102, 540–545. 10.1094/PDIS-04-17-0517-RE 30673478

[B53] Ng P. C. Henikoff S. (2001). Predicting deleterious amino acid substitutions. Genome Res. 11, 863–874. 10.1101/gr.176601 11337480 PMC311071

[B54] Nuskern L. Tkalec M. Ježić M. Katanić Z. Krstin L. Curković-Perica M. (2017). *Cryphonectria hypovirus* 1-induced changes of stress enzyme activity in transfected phytopathogenic fungus *Cryphonectria parasitica*. Microb. Ecol. 74, 302–311. 10.1007/s00248-017-0945-7 28160056

[B55] Nuss D. L. (2005). Hypovirulence: mycoviruses at the fungal–plant interface. Nat. Rev. Microbiol. 3, 632–642. 10.1038/nrmicro1206 16064055

[B56] Peever T. L. Liu Y. C. Wang K. Hillman B. I. Foglia R. Milgroom M. G. (1998). Incidence and diversity of double-stranded RNAs occurring in the chestnut blight fungus, *Cryphonectria parasitica*, in China and Japan. Phytopathology 88, 811–817. 10.1094/PHYTO.1998.88.8.811 18944887

[B57] Peruski A. H. Peruski L. F. (2003). Immunological methods for detection and identification of infectious disease and biological warfare agents. Clin. Diagn. Lab. Immunol. 10, 506–513. 10.1128/CDLI.10.4.506-513.2003 12853377 PMC164256

[B58] Peters F. S. Bußkamp J. Prospero S. Rigling D. Metzler B. (2014). Genetic diversification of the chestnut blight fungus *Cryphonectria parasitica* and its associated hypovirus in Germany. Fungal Biol. 118, 193–210. 10.1016/j.funbio.2013.11.009 24528641

[B59] Pettersen E. F. Goddard T. D. Huang C. C. Couch G. S. Greenblatt D. M. Meng E. C.. (2004). UCSF Chimera– A visualization system for exploratory research and analysis. J. Comput. Chem. 25, 1605–1612. 10.1002/jcc.20084 15264254

[B60] Pettersen E. F. Goddard T. D. Huang C. C. Meng E. C. Couch G. S. Croll T. I.. (2021). UCSF Chimera: structure visualization for researchers, educators, and developers. Prot. Sci. 30, 70–82. 10.1002/pro.3943 32881101 PMC7737788

[B61] Prospero S. Conedera M. Heiniger U. Rigling D. (2006). Saprophytic activity and sporulation of *Cryphonectria parasitica* on dead chestnut wood in forests with naturally established hypovirulence. Phytopathology 96, 1337–1344. 10.1094/PHYTO-96-1337 18943666

[B62] Prospero S. Rigling D. (2016). Using molecular markers to assess the establishment and spread of a mycovirus applied as a biological control agent against chestnut blight. Biocontrol 61, 313–323. 10.1007/s10526-015-9713-0

[B63] Rigling D. Borst N. Cornejo C. Supatashvili A. Prospero S. (2018). Genetic and phenotypic characterization of *Cryphonectria hypovirus* 1 from Eurasian Georgia. Viruses 10:687. 10.3390/v10120687 30513977 PMC6315935

[B64] Rigling D. Prospero S. (2018). *Cryphonectria parasitica*, the causal agent of chestnut blight: invasion history, population biology and disease control. Mol. Plant Pathol. 19, 7–20. 10.1111/mpp.12542 28142223 PMC6638123

[B65] Roane M. K. Griffin G. J. Elkins J. R. (1986). Chestnut Blight, Other Endothia Diseases, and the Genus Endothia. St. Paul, MN: The American Phytopathological Society.

[B66] Robin C. Anziani C. Cortesi P. (2000). Relationship between biological control, incidence of hypovirulence, and diversity of vegetative compatibility types of *Cryphonectria parasitica* in France. Phytopathology 90, 730–737. 10.1094/PHYTO.2000.90.7.730 18944492

[B67] Robin C. Lanz S. Soutrenon A. Rigling D. (2010). Dominance of natural over released biological control agents of the chestnut blight fungus *Cryphonectria parasitica* in south-eastern France is associated with fitness-related traits. Biol. Control 53, 55–61. 10.1016/j.biocontrol.2009.10.013

[B68] Romon-Ochoa P. Forster J. Chitty R. Gorton C. Lewis A. Eacock A.. (2022). Canker development and biocontrol potential of CHV-1 infected English isolates of *Cryphonectria parasitica* is dependent on the virus concentration and the compatibility of the fungal inoculums. Viruses 14:2678. 10.3390/v14122678 36560682 PMC9785502

[B69] Romon-Ochoa P. Gorton C. Lewis A. van der Linde S. Webber J. Pérez-Sierra A. (2020). Hypovirulent effect of the *Cryphonectria hypovirus* 1 in British isolates of *Cryphonectria parasitica*. Pest Manag. Sci. 76, 1333–1343. 10.1002/ps.5644 31603609

[B70] Roth C. Liberles D. A. (2006). A systematic search for positive selection in higher plants (Embryophytes). BMC Plant Biol. 6, 1–11. 10.1186/1471-2229-6-12 16784532 PMC1540423

[B71] Rozas J. Ferrer-Mata A. Sánchez-DelBarrio J. C. Guirao-Rico S. Librado P. Ramos-Onsins S. E.. (2017). DnaSP 6: DNA sequence polymorphism analysis of large data sets. Mol. Biol. Evol. 34, 3299–3302. 10.1093/molbev/msx248 29029172

[B72] Shapovalov M. V. Dunbrack R. L. (2011). A smoothed backbone-dependent rotamer library for proteins derived from adaptive kernel density estimates and regressions. Structure 19, 844–858. 10.1016/j.str.2011.03.019 21645855 PMC3118414

[B73] Sim N. L. Kumar P. Hu J. Henikoff S. Schneider G. Ng P. C. (2012). SIFT web server: predicting effects of amino acid substitutions on proteins. Nucleic Acids Res. 40, W452–W457. 10.1093/nar/gks539 22689647 PMC3394338

[B74] Sotirovski K. Milgroom M. G. Rigling D. Heiniger U. (2006). Occurrence of *Cryphonectria hypovirus* 1 in the chestnut blight fungus in Macedonia. For. Pathol. 36, 136–143. 10.1111/j.1439-0329.2006.00443.x

[B75] Sotirovski K. Rigling D. Heiniger U. Milgroom M. G. (2011). Variation in virulence of *Cryphonectria hypovirus* 1 in Macedonia. For. Pathol. 41, 59–65. 10.1111/j.1439-0329.2009.00637.x

[B76] Stauber L. Croll D. Prospero S. (2022). Temporal changes in pathogen diversity in a perennial plant–pathogen–hyperparasite system. Mol. Ecol. 31, 2073–2088. 10.1111/mec.16386 35122694 PMC9540319

[B77] Suzuki N. Ghabrial S. A. Kim K. H. Pearson M. Marzano S. Y. L. Yaegashi H.. (2018). ICTV virus taxonomy profile: Hypoviridae. J. Gen. Virol. 99, 615–616. 10.1099/jgv.0.001055 29589826 PMC12662185

[B78] Suzuki N. Maruyama K. Moriyama M. Nuss D. L. (2003). Hypovirus papain-like protease p29 functions in trans to enhance viral double-stranded RNA accumulation and vertical transmission. J. Virol. 77, 11697–11707. 10.1128/JVI.77.21.11697-11707.2003 14557655 PMC229363

[B79] Suzuki N. Nuss D. L. (2002). Contribution of protein p40 to hypovirus-mediated modulation of fungal host phenotype and viral RNA accumulation. J. Virol. 76, 7747–7759. 10.1128/JVI.76.15.7747-7759.2002 12097588 PMC136391

[B80] Tamura K. Nei M. (1993). Estimation of the number of nucleotide substitutions in the control region of mitochondrial DNA in humans and chimpanzees. Mol. Biol. Evol. 10, 512–526. 8336541 10.1093/oxfordjournals.molbev.a040023

[B81] Tsetsarkin K. A. Vanlandingham D. L. McGee C. E. Higgs S. (2007). A single mutation in chikungunya virus affects vector specificity and epidemic potential. PLoS Pathog. 3:e201. 10.1371/journal.ppat.0030201 18069894 PMC2134949

[B82] Turina M. Rostagno L. (2007). Virus-induced hypovirulence in *Cryphonectria parasitica*: still an unresolved conundrum. J. Plant Pathol. 89, 165–178. Available online at: https://www.jstor.org/stable/41998375

[B83] Waldboth M. Oberhuber W. (2009). Synergistic effect of drought and chestnut blight (*Cryphonectria parasitica*) on growth decline of European chestnut (*Castanea sativa*). For. Pathol. 39, 43–55. 10.1111/j.1439-0329.2008.00562.x

[B84] Waterhouse A. M. Procter J. B. Martin D. M. A. Clamp M. Barton G. J. (2009). Jalview Version 2—a multiple sequence alignment editor and analysis workbench. Bioinformatics 25, 1189–1191. 10.1093/bioinformatics/btp033 19151095 PMC2672624

[B85] Wickham H. (2016). ggplot2: Elegant Graphics for Data Analysis. New York, NY: Springer-Verlag. Available online at: https://ggplot2.tidyverse.org (accessed January 15, 2024).

[B86] Yaegashi H. Sawahata T. Ito T. Kanematsu S. (2011). A novel colony-print immunoassay reveals differential patterns of distribution and horizontal transmission of four unrelated mycoviruses in *Rosellinia necatrix*. Virology 409, 280–289. 10.1016/j.virol.2010.10.014 21056891

[B87] Yates C. M. Sternberg M. J. E. (2013). The effects of non-synonymous single nucleotide polymorphisms (nsSNPs) on protein–protein interactions. J. Mol. Biol. 425, 3949–3963. 10.1016/j.jmb.2013.07.012 23867278

[B88] Zamora P. Martín A. B. Rigling D. Diez J. J. (2012). Diversity of *Cryphonectria parasitica* in western Spain and identification of hypovirus-infected isolates. For. Pathol. 42, 412–419. 10.1111/j.1439-0329.2012.00775.x

[B89] Zamora P. Rigling D. Diez J. J. (2007). Detection of hypovirulent isolates of *Cryphonectria parasitica* in Castilla y Leon, Spain, in Proceedings of the II Iberian Congress on Chestnut 784, 163–168. 10.17660/ActaHortic.2008.784.25

