# Supplementary material for: Molecular genetic variability of Cryphonectria hypovirus 1 associated with Cryphonectria parasitica in South Tyrol (northern Italy)
Source: Front Microbiol. 2024 Feb 27;15:1291542. doi: 10.3389/fmicb.2024.1291542 (PMC10927965; doi:10.3389/fmicb.2024.1291542)
Supplement: Supplementary file 1 [file Data_Sheet_1.PDF]

Supplementary Material

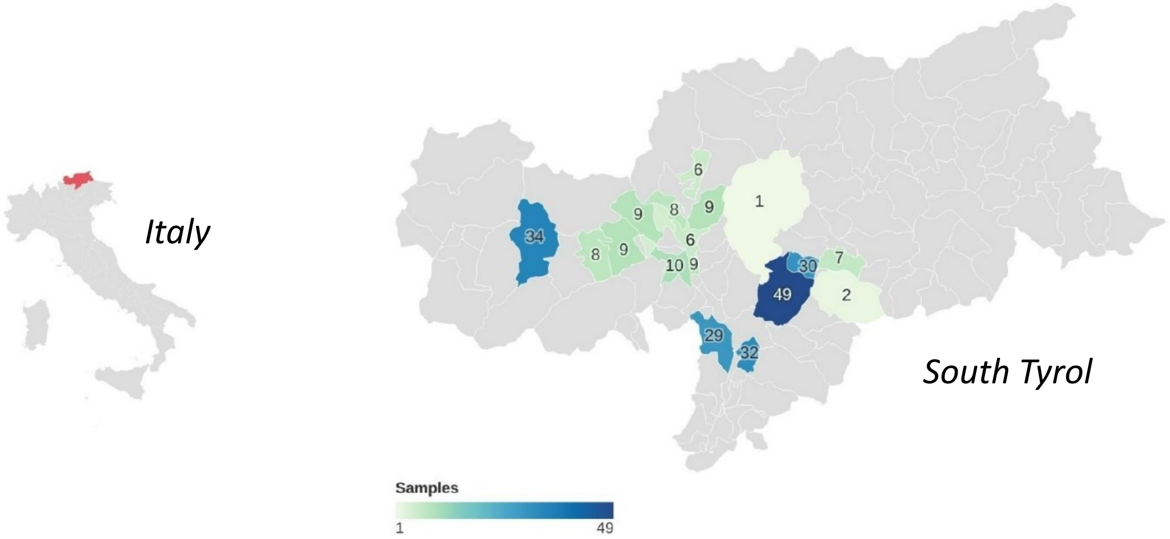

Supplementary Figure 1. Map of Italy with highlighted study area of South Tyrol (left). Zoomed map of South Tyrol, in which municipalities are highlighted where samples of *Cryphonectria parasitica* were collected. The numbers denote the number of isolates obtained from each municipality (right).

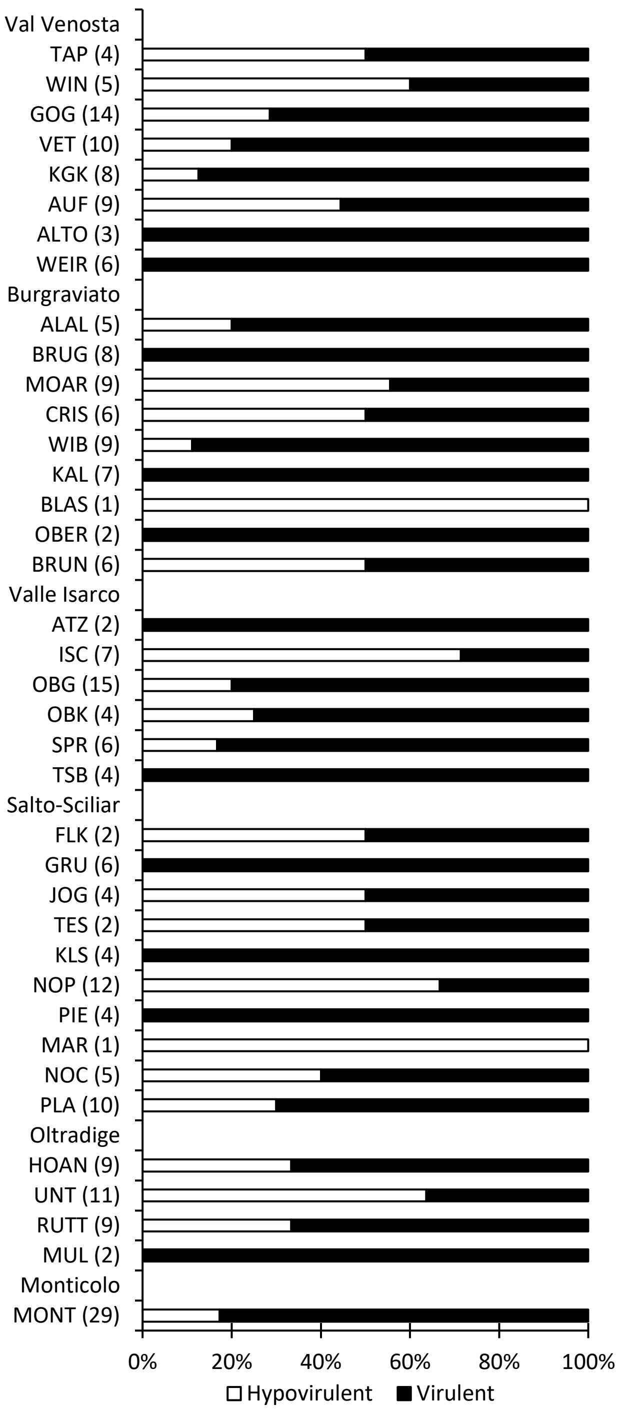

Supplementary Figure 2. Frequency of *Cryphonectria hypovirus* 1 (CHV-1) present in the isolates of *Cryphonectria parasitica* in different chestnut stands of South Tyrol. CHV-1 containing isolates are named as hypovirulent (white bars), whereas isolates without CHV-1 are referred to as virulent (black bars). Localities of chestnut stands, their districts and the number of samples are mentioned in the Supplementary Table 1.

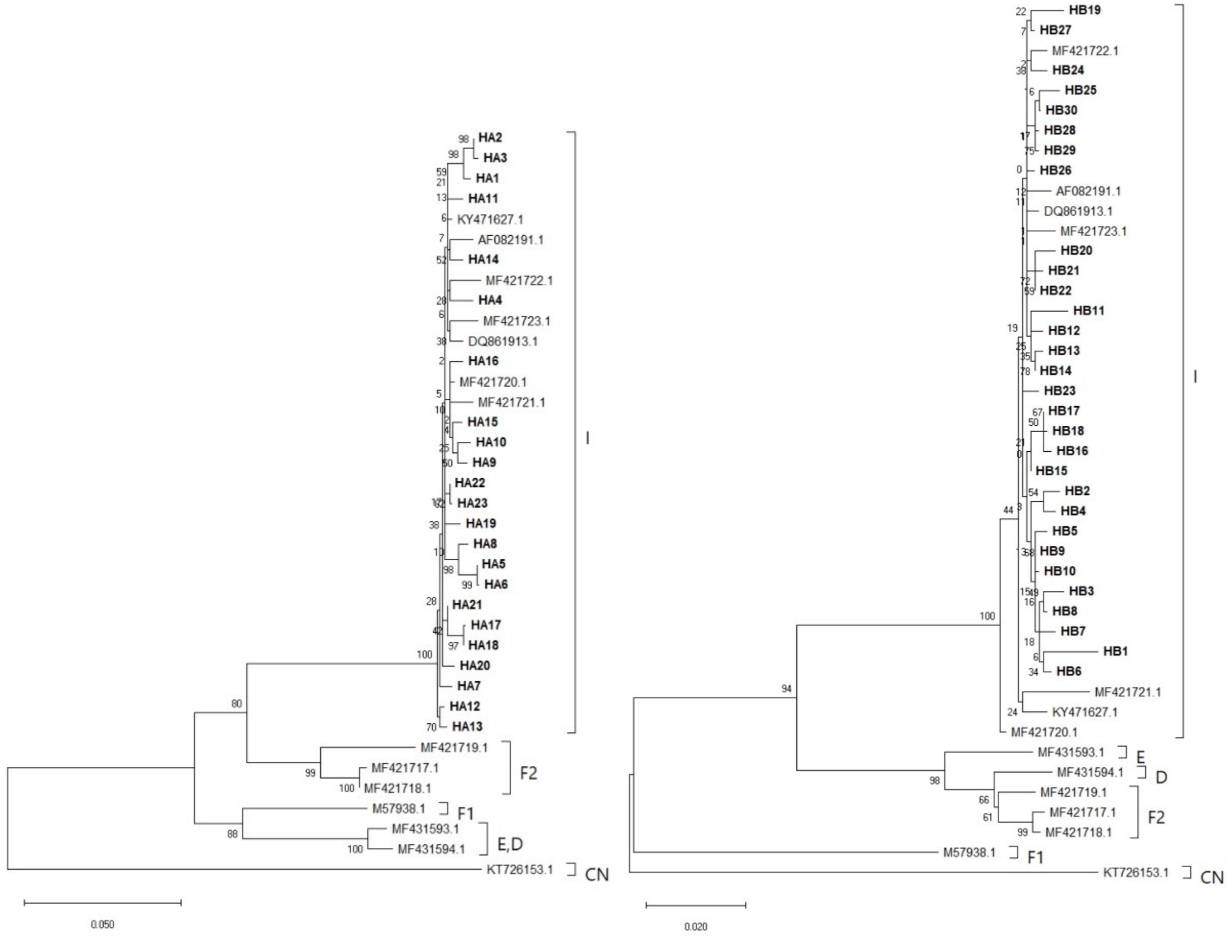

ORF-A ORF-B

Supplementary Figure 3. Maximum likelihood trees based on partial sequences of Open Reading Frame (ORF)-A and ORF-B. The haplotypes of CHV-1 from South Tyrol are indicated with their numbers, whereas sequences from GenBank are labelled with their accession numbers. CHV-1 subtypes are indicated with letters: CN, Chinese; D, German; E, Spanish; F1, F2, French; I, Italian. Sequences obtained from South Tyrol (this study) are highlighted in bold.

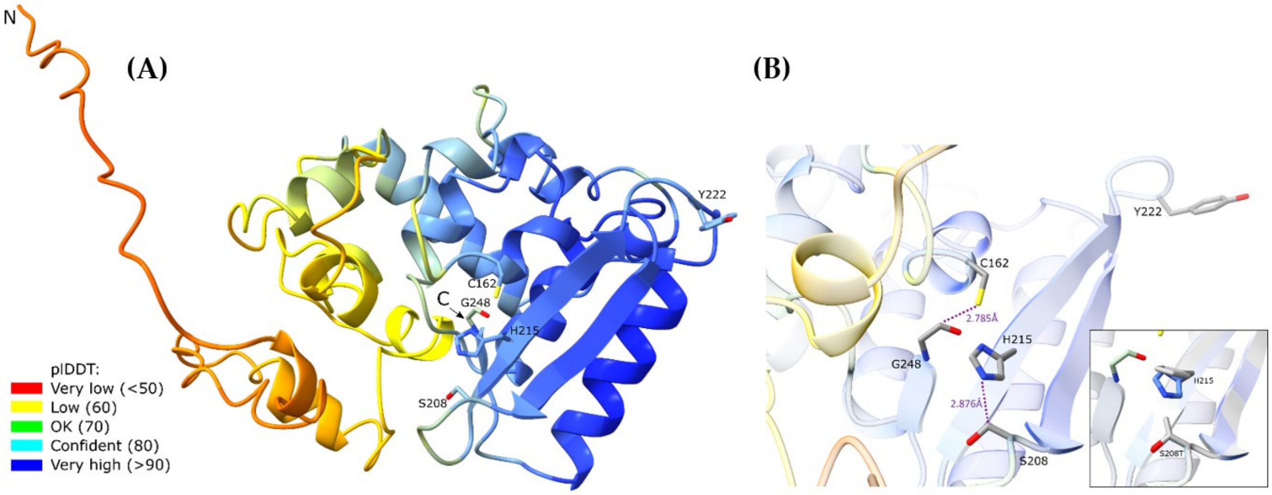

Supplementary Figure 4. A computed structure model of p29 of CHV-1 EP721 generated by AlphaFold2. (A) The full-length structure of p29 (248 amino acids) is depicted with colour-coded confidence scores: very low (red), low (yellow), OK (green), confident (cyan), and very high (blue). Catalytic residues (Cys162 and His215), cleavage site residue (Gly248), and mutated residues (Ser208 and Tyr222) are represented as sticks. (B) A close-up of the p29 active site. The distance between the residues that are close enough to clash or make contact is shown. The inset figure presents an overlay of p29 wildtype and Ser208Thr models (colored in gray). After energy-minimization, the conformation of His215 is changed and the clash is relieved.

Supplementary Table 1. Number of fungal isolates used in this study and their sampling sites, including the localities and the districts.

District	No.	Code of chestnut stand	Locality (German name – Italian name)	Number of isolates
Vinschgau – Val Venosta				
	1	TAP	Kortsch – Corces	4
	2	WIN	Kortsch – Corces	5
	3	GOG	Schlanders – Silandro	15
	4	VET	Schlanders – Silandro	10
	5	KGK	Kastelbell – Ciardes	8
	6	AUF	Naturns – Naturno	9
	7	ALTO	Töll – Tel	3
	8	WEIR	Töll – Tel	6
Burggrafenamt – Burgraviato				
	9	ALAL	Alt Algund – Lagundo	5
	10	BRUG	Dorf Tirol – Tirolo	8
	11	MOAR	Schenna – Scena	9
	12	CRIS	Meran – Merano	6
	13	WIB	Burgstall – Postal	9
	14	KAL	Lana	7
	15	BLAS	Völlan – Foiana	1
	16	OBER	Völlan – Foiana	2
	17	BRUN	Saltaus – Saltusio	6
Eisacktal – Valle Isarco				
	18	ATZ	Atzwang- Campodazzo	2
	19	ISC	Lajen – Laion	7
	20	OBG	Barbian – Barbiano	16
	21	OBK	Barbian – Barbiano	4
	22	SPR	Barbian – Barbiano	6
	23	TSB	Barbian – Barbiano	4
Salten-Schlern – Salto-Sciliar				
	24	FLK	St. Oswald-Seis – Sant'Osvaldo-Siusi	2
	25	GRU	Rotwand-Ritten – Pietrarossa-Renon	6
	26	JOG	Siffian-Ritten – Siffiano-Renon	4
	27	TES	Unterinn-Ritten – Auna di Sotto-Renon	2
	28	KLS	Signat-Ritten – Signato-Renon	4
	29	NOP	Lengstein-Ritten – Longostano-Renon	12
	30	PIE	Lengstein-Ritten – Longostano-Renon	4
	31	MAR	Sarntal- Sarentino	1
	32	NOC	Sill-Ritten – Sill-Renon	5
	33	PLA	Sill-Ritten – Sill-Renon	10
Überetsch-Unterland – Oltradige-Bassa Atesina				
	34	HOAN	Leifers – Laives	9
	35	UNT	Leifers – Laives	11
	36	RUTT	Steinmannwald – Pineta	9
	37	MUL	Steinmannwald – Pineta	3
Monticolo Forest				
	38	Mont	Montiggler Wald – Bosco di Monticolo	29

Supplementary Table 2. Primers used in this study to amplify and sequence open reading frames (ORFs) of *Cryphonectria hypovirus* 1.

Target gene	Primer name	Sequence (5' to 3')	Amplicon length (kbp)	References
ORF-A	EP713-5	TTG GAT TCC CGA TTC CTT CA	1.7	Allemann *et al.* [43]
	R2280	CTT ACC ATC TCG CTT GAC AAA		
	Hvep-1*	TGA CAC GGA AGC TGA GTG TC	-	Gobbin *et al.* [30]
	Hvep-2*	AGC GCG AAT TTC TTG TCG		
ORF-B	EP713-6	GCC ACT TCG TGG GAC AAT AC	1.5	Allemann *et al.* [43]
	EP713-7	GGG AAG AAA GTG GAC TTA CC		

* Primers only used for Sanger sequencing

Supplementary Table 3. Complete dataset of all isolates and their assessment using phenotypic characterization, the dot-blot immunoassay implemented in this study and RT-PCR.

No.	Isolate code	Phenotypic assessment	Sample code immunoassay	Result immunoassay*	Result RT-PCR ORF-A	Result RT-PCR ORF-B	Classification of isolate
1	CP01-01	Intermediate	CPi12-46	dsRNA absent (0/3)	-	-	Virulent
2	CP01-02	Hypovirulent	CPi11-41	dsRNA present (3/3)	Amplicon present	Amplicon present	Hypovirulent
3	CP01-06	Intermediate	CPi14-12	dsRNA absent (0/3)	-	-	Virulent
4	CP01-11	Hypovirulent	CPi7-11	dsRNA absent (0/3)	-	-	Virulent
5	CP01-12	Intermediate	CPi14-13	dsRNA absent (0/3)	-	-	Virulent
6	CP01-13	Virulent	CPi12-05	dsRNA absent (0/3)	-	-	Virulent
7	CP01-17	Hypovirulent	CPi10-16	dsRNA absent (0/3)	-	-	Virulent
8	CP01-19	Hypovirulent	CPi9-06	dsRNA present (3/3)	Amplicon present	Amplicon present	Hypovirulent
9	CP02-01	Intermediate	CPi9-10	dsRNA absent (0/3)	-	-	Virulent
10	CP02-03	Intermediate	CPi7-08	dsRNA absent (0/3)	-	-	Virulent
11	CP02-05	Intermediate	CPi14-14	dsRNA absent (0/3)	-	-	Virulent
12	CP02-06	Hypovirulent	CPi9-08	dsRNA present (3/3)	Amplicon present	Amplicon present	Hypovirulent
13	CP02-09	Hypovirulent	CPi9-05	dsRNA present (3/3)	Amplicon present	Amplicon present	Hypovirulent
14	CP02-11	Hypovirulent	CPi9-03	dsRNA present (3/3)	Amplicon present	Amplicon present	Hypovirulent
15	CP02-12	Intermediate	CPi10-42	dsRNA absent (0/3)	No amplicon	No amplicon	Virulent
16	CP02-15	Hypovirulent	CPi11-42	dsRNA present (3/3)	Amplicon present	Amplicon present	Hypovirulent
17	CP02-16	Hypovirulent	CPi14-16	dsRNA present (2/3)	Amplicon present	Amplicon present	Hypovirulent
18	CP02-17	Hypovirulent	CPi14-29	dsRNA present (3/3)	Amplicon present	Amplicon present	Hypovirulent
19	CP02-18	Hypovirulent	CPI8-03	dsRNA absent (0/3)	No amplicon	No amplicon	Virulent
20	CP04-17	Hypovirulent	CPi15-01	dsRNA present (3/3)	No amplicon	No amplicon	Virulent
21	CP06-01	Intermediate	CPi14-10	dsRNA absent (0/3)	-	-	Virulent
22	CP06-02	Intermediate	CPi14-11	dsRNA absent (0/3)	-	-	Virulent
23	CP06-03	Hypovirulent	CPi14-15	dsRNA present (3/3)	Amplicon present	No amplicon	Hypovirulent
24	CP09-05	Hypovirulent	CPi15-21	dsRNA present (3/3)	No amplicon	No amplicon	Virulent
25	CP09-06	Hypovirulent	CPi15-28	dsRNA present (3/3)	Amplicon present	No amplicon	Hypovirulent
26	CP09-07	Intermediate	CPi11-23	dsRNA absent (0/3)	-	-	Virulent
27	CP09-08	Intermediate	CPi11-47	dsRNA absent (0/3)	-	-	Virulent
28	CP09-09	Intermediate	CPi14-02	dsRNA present (3/3)	Amplicon present	Amplicon present	Hypovirulent
29	CP09-10	Virulent	CPi7-15	dsRNA absent (0/3)	-	-	Virulent
30	CP09-11	Intermediate	CPi14-17	dsRNA absent (0/3)	-	-	Virulent
31	CP09-12	Intermediate	CPi15-02	dsRNA absent (0/3)	-	-	Virulent
32	CP09-13	Intermediate	CPi12-15	dsRNA absent (0/3)	-	-	Virulent
33	CP09-14	Hypovirulent	CPi8-01	dsRNA absent (0/3)	-	-	Virulent
34	CP09-15	Hypovirulent	CPi14-30	dsRNA present (3/3)	No amplicon	Amplicon present	Hypovirulent
35	CP09-16	Intermediate	CPi14-04	dsRNA present (1/3)	Amplicon present	Amplicon present	Hypovirulent
36	CP09-17	Virulent	CPi15-03	dsRNA absent (0/3)	-	-	Virulent
37	CP09-18	Hypovirulent	CPi14-09	dsRNA absent (0/3)	Amplicon present	Amplicon present	Hypovirulent
38	CP09-19	Intermediate	CPi15-11	dsRNA absent (0/3)	-	-	Virulent
39	CP09-20	Virulent	CPi15-12	dsRNA absent (0/3)	-	-	Virulent
40	CP10-01	Virulent	CPi15-32	dsRNA absent (0/3)	-	-	Virulent
41	CP10-04	Intermediate	CPi15-33	dsRNA absent (0/3)	-	-	Virulent
42	CP10-07	Hypovirulent	CPi14-18	dsRNA present (3/3)	No amplicon	Amplicon present	Hypovirulent
43	CP10-09	Hypovirulent	CPi14-19	dsRNA present (3/3)	Amplicon present	Amplicon present	Hypovirulent
44	CP10-10	Hypovirulent	CPi14-20	dsRNA present (3/3)	No amplicon	No amplicon	Virulent
45	CP10-11	Intermediate	CPi15-16	dsRNA absent (0/3)	-	-	Virulent
46	CP10-12	Hypovirulent	CPi14-06	dsRNA absent (0/3)	-	-	Virulent
47	CP10-14	Hypovirulent	CPi15-15	dsRNA present (3/3)	Amplicon present	No amplicon	Hypovirulent
48	CP10-15	Intermediate	CPi10-31	dsRNA absent (0/3)	-	-	Virulent
49	CP10-16	Intermediate	CPi14-03	dsRNA present (3/3)	Amplicon present	Amplicon present	Hypovirulent
50	CP10-18	Hypovirulent	CPi7-04	dsRNA present (2/3)	Amplicon present	Amplicon present	Hypovirulent
51	CP10-19	Intermediate	CPi15-34	dsRNA present (3/3)	Amplicon present	Amplicon present	Hypovirulent
52	CP10-20	Virulent	Lost to contamination				
53	CP11-03	Intermediate	CPi15-17	dsRNA absent (0/3)	-	-	Virulent
54	CP11-05	Virulent	CPi15-18	dsRNA absent (0/3)	-	-	Virulent
55	CP11-06	Intermediate	CPi10-13	dsRNA present(3/3)	Amplicon present	Amplicon present	Hypovirulent
56	CP11-07	Hypovirulent	CPi11-02	dsRNA present (3/3)	No amplicon	No amplicon	Virulent
57	CP11-08	Intermediate	CPi15-19	dsRNA absent (0/3)	-	-	Virulent

No.	Isolate code	Phenotypic assessment	Sample code immunoassay	Result immunoassay*	Result RT-PCR ORF-A	Result RT-PCR ORF-B	Classification of isolate
58	CP11-10	Intermediate	CPi15-31	dsRNA absent (0/3)	-	-	Virulent
59	CP11-12	Intermediate	CPi15-13	dsRNA absent (0/3)	-	-	Virulent
60	CP11-15	Intermediate	CPi15-14	dsRNA absent (0/3)	Amplicon present	No amplicon	Hypovirulent
61	CP11-18	Hypovirulent	CPi11-40	dsRNA present (3/3)	Amplicon present	Amplicon present	Hypovirulent
62	CP11-19	Hypovirulent	CPi11-45	dsRNA present (3/3)	Amplicon present	Amplicon present	Hypovirulent
63	CP11-20	Intermediate	CPi15-20	dsRNA absent (0/3)	-	-	Virulent
64	CP12-01	Intermediate	CPi7-22	dsRNA absent (0/3)	Amplicon present	No amplicon	Hypovirulent
65	CP12-02	Intermediate	CPi7-19	dsRNA absent (0/3)	-	-	Virulent
66	CP12-03	Intermediate	CPi11-32	dsRNA absent (0/3)	-	-	Virulent
67	CP12-04	Intermediate	CPi10-19	dsRNA absent (0/3)	-	-	Virulent
68	CP12-05	Intermediate	CPi15-22	dsRNA absent (0/3)	-	-	Virulent
69	CP12-06	Intermediate	CPi12-14	dsRNA absent (0/3)	No amplicon	No amplicon	Virulent
70	CP12-07	Intermediate	CPi12-43	dsRNA absent (0/3)	No amplicon	No amplicon	Virulent
71	CP12-08	Virulent	CPi15-23	dsRNA absent (0/3)	-	-	Virulent
72	CP12-09	Intermediate	CPi12-32	dsRNA absent (0/3)	-	-	Virulent
73	CP12-10	Intermediate	CPi12-45	dsRNA present (2/3)	Amplicon present	Amplicon present	Hypovirulent
74	CP12-11	Intermediate	CPi11-48	dsRNA absent (0/3)	-	-	Virulent
75	CP12-12	Intermediate	CPi11-14	dsRNA absent (0/3)	-	-	Virulent
76	CP12-13	Intermediate	CPi12-28	dsRNA absent (0/3)	-	-	Virulent
77	CP12-14	Intermediate	CPi10-09	dsRNA absent (0/3)	-	-	Virulent
78	CP12-15	Intermediate	CPi12-29	dsRNA absent (0/3)	-	-	Virulent
79	CP12-16	Intermediate	CPi12-38	dsRNA absent (0/3)	No amplicon	No amplicon	Virulent
80	CP12-17	Intermediate	CPi10-24	dsRNA absent (0/3)	-	-	Virulent
81	CP12-18	Hypovirulent	CPi9-15	dsRNA present (3/3)	Amplicon present	Amplicon present	Hypovirulent
82	CP12-19	Intermediate	CPi12-44	dsRNA absent (0/3)	-	-	Virulent
83	CP12-20	Intermediate	CPi11-25	dsRNA absent (0/3)	-	-	Virulent
84	CP12-21	Intermediate	CPi12-16	dsRNA absent (0/3)	-	-	Virulent
85	CP12-22	Intermediate	CPi10-18	dsRNA absent (0/3)	-	-	Virulent
86	CP12-23	Intermediate	CPi11-46	dsRNA absent (0/3)	No amplicon	No amplicon	Virulent
87	CP12-24	Intermediate	CPi12-25	dsRNA absent (0/3)	-	-	Virulent
88	CP12-25	Intermediate	CPi15-24	dsRNA absent (0/3)	-	-	Virulent
89	CP12-26	Intermediate	CPi10-36	dsRNA absent (0/3)	-	-	Virulent
90	CP12-27	Intermediate	CPi14-08	dsRNA absent (0/3)	-	-	Virulent
91	CP12-28	Hypovirulent	CPi10-27	dsRNA absent (0/3)	-	-	Virulent
92	CP13-04	Hypovirulent	CPi14-05	dsRNA absent (0/3)	-	-	Virulent
93	CP13-05	Hypovirulent	Lost to contamination				
94	CP13-06	Hypovirulent	Lost to contamination				
95	CP14-01	Intermediate	CPi12-09	dsRNA absent (0/3)	-	-	Virulent
96	CP14-02	Intermediate	CPi7-14	dsRNA absent (0/3)	-	-	Virulent
97	CP14-03	Intermediate	CPi9-23	dsRNA absent (0/3)	-	-	Virulent
98	CP14-04	Intermediate	CPi12-18	dsRNA absent (0/3)	-	-	Virulent
99	CP14-05	Hypovirulent	CPi11-39	dsRNA present (3/3)	No amplicon	No amplicon	Virulent
100	CP14-06	Hypovirulent	CPi7-03	dsRNA present (3/3)	Amplicon present	Amplicon present	Hypovirulent
101	CP14-07	Virulent	CPi10-10	dsRNA absent (0/3)	-	-	Virulent
102	CP14-08	Hypovirulent	CPi11-05	dsRNA present (3/3)	Amplicon present	Amplicon present	Hypovirulent
103	CP14-09	Hypovirulent	CPi11-03	dsRNA present (3/3)	No amplicon	Amplicon present	Hypovirulent
104	CP14-10	Hypovirulent	CPi10-02	dsRNA present (3/3)	Amplicon present	Amplicon present	Hypovirulent
105	CP14-11	Virulent	CPi12-33	dsRNA absent (0/3)	-	-	Virulent
106	CP14-12	Intermediate	CPi12-37	dsRNA absent (0/3)	No amplicon	No amplicon	Virulent
107	CP14-13	Intermediate	CPi10-01	dsRNA absent (0/3)	-	-	Virulent
108	CP14-14	Intermediate	CPi10-06	dsRNA absent (0/3)	No amplicon	No amplicon	Virulent
109	CP14-15	Intermediate	CPi10-08	dsRNA absent (0/3)	-	-	Virulent
110	CP14-16	Intermediate	CPi10-30	dsRNA absent (0/3)	No amplicon	No amplicon	Virulent
111	CP14-17	Hypovirulent	CPi10-44	dsRNA present (3/3)	Amplicon present	Amplicon present	Hypovirulent
112	CP14-18	Intermediate	CPi12-42	dsRNA present (3/3)	Amplicon present	Amplicon present	Hypovirulent
113	CP14-20	Intermediate	CPi12-08	dsRNA absent (0/3)	No amplicon	No amplicon	Virulent
114	CP14-21	Intermediate	CPi14-21	dsRNA absent (0/3)	-	-	Virulent
115	CP14-22	Intermediate	CPi14-22	dsRNA absent (0/3)	-	-	Virulent
116	CP14-23	Hypovirulent	CPi9-07	dsRNA present (3/3)	Amplicon present	Amplicon present	Hypovirulent
117	CP15-01	Hypovirulent	CPi11-06	dsRNA absent (0/3)	-	-	Virulent
118	CP15-02	Hypovirulent	CPi11-07	dsRNA present (3/3)	Amplicon present	Amplicon present	Hypovirulent

No.	Isolate code	Phenotypic assessment	Sample code immunoassay	Result immunoassay*	Result RT-PCR ORF-A	Result RT-PCR ORF-B	Classification of isolate
119	CP15-03	Intermediate	CPi11-29	dsRNA absent (0/3)	-	-	Virulent
120	CP15-04	Intermediate	CPi14-23	dsRNA absent (0/3)	-	-	Virulent
121	CP15-05	Intermediate	CPi7-21	dsRNA absent (0/3)	Amplicon present	Amplicon present	Hypovirulent
122	CP15-06	Hypovirulent	CPi10-43	dsRNA present (3/3)	Amplicon present	Amplicon present	Hypovirulent
123	CP15-07	Hypovirulent	CPi11-44	dsRNA present (3/3)	Amplicon present	Amplicon present	Hypovirulent
124	CP15-08	Intermediate	CPi14-24	dsRNA absent (0/3)	-	-	Virulent
125	CP15-09	Hypovirulent	CPi9-11	dsRNA absent (0/3)	-	-	Virulent
126	CP15-10	Intermediate	CPi11-09	dsRNA absent (0/3)	No amplicon	No amplicon	Virulent
127	CP15-11	Intermediate	CPi11-08	dsRNA absent (0/3)	-	-	Virulent
128	CP15-12	Intermediate	CPi14-07	dsRNA absent (0/3)	-	-	Virulent
129	CP15-13	Hypovirulent	CPi10-05	dsRNA present (3/3)	Amplicon present	Amplicon present	Hypovirulent
130	CP15-14	Hypovirulent	CPi13-04	dsRNA present (3/3)	Amplicon present	Amplicon present	Hypovirulent
131	CP15-15	Intermediate	CPi10-12	dsRNA present (3/3)	Amplicon present	Amplicon present	Hypovirulent
132	CP15-16	Hypovirulent	CPi10-14	dsRNA present (3/3)	No amplicon	No amplicon	Virulent
133	CP15-17	Intermediate	CPi11-43	dsRNA absent (0/3)	No amplicon	No amplicon	Virulent
134	CP16-01	Intermediate	CPi11-38	dsRNA present (1/3)	Amplicon present	Amplicon present	Hypovirulent
135	CP16-02	Hypovirulent	CPi8-16	dsRNA present (3/3)	Amplicon present	Amplicon present	Hypovirulent
136	CP16-03	Intermediate	CPi7-05	dsRNA present (3/3)	Amplicon present	Amplicon present	Hypovirulent
137	CP16-04	Intermediate	CPi14-25	dsRNA absent (0/3)	-	-	Virulent
138	CP17-01	Intermediate	CPi14-26	dsRNA absent (0/3)	-	-	Virulent
139	CP17-05	Intermediate	CPi14-27	dsRNA absent (0/3)	-	-	Virulent
140	CP19-01	Intermediate	CPi12-30	dsRNA absent (0/3)	No amplicon	No amplicon	Virulent
141	CP19-02	Virulent	CPi14-28	dsRNA absent (0/3)	-	-	Virulent
142	CP19-03	Hypovirulent	CPi7-07	dsRNA present (1/3)	Amplicon present	Amplicon present	Hypovirulent
143	CP19-05	Intermediate	CPi15-30	dsRNA absent (0/3)	-	-	Virulent
144	CP20-01	Hypovirulent	CPi8-18	dsRNA present (3/3)	Amplicon present	Amplicon present	Hypovirulent
145	CP20-02	Hypovirulent	CPi8-19	dsRNA absent (0/3)	-	-	Virulent
146	CP20-03	Hypovirulent	CPi8-20	dsRNA present (3/3)	Amplicon present	Amplicon present	Hypovirulent
147	CP20-04	Intermediate	CPi12-26	dsRNA absent (0/3)	-	-	Virulent
148	CP20-05	Intermediate	CPi11-22	dsRNA absent (0/3)	-	-	Virulent
149	CP20-06	Intermediate	CPi11-27	dsRNA absent (0/3)	-	-	Virulent
150	CP20-07	Hypovirulent	CPi8-15	dsRNA absent (0/3)	-	-	Virulent
151	CP20-08	Hypovirulent	CPi12-35	dsRNA present (3/3)	Amplicon present	Amplicon present	Hypovirulent
152	CP20-10	Hypovirulent	CPi8-04	dsRNA present (3/3)	Amplicon present	Amplicon present	Hypovirulent
153	CP20-11	Virulent	CPi8-22	dsRNA absent (0/3)	-	-	Virulent
154	CP20-12	Hypovirulent	CPi8-05	dsRNA present (3/3)	Amplicon present	Amplicon present	Hypovirulent
155	CP20-13	Virulent	CPi10-34	dsRNA absent (0/3)	-	-	Virulent
156	CP20-14	Intermediate	CPi10-25	dsRNA absent (0/3)	-	-	Virulent
157	CP21-01	Intermediate	CPi10-17	dsRNA absent (0/3)	-	-	Virulent
158	CP21-02	Hypovirulent	CPi8-10	dsRNA present (2/3)	Amplicon present	Amplicon present	Hypovirulent
159	CP21-03	Intermediate	CPi10-29	dsRNA absent (0/3)	-	-	Virulent
160	CP21-04	Virulent	CPi15-29	dsRNA absent (0/3)	-	-	Virulent
161	CP21-05	Hypovirulent	CPi9-02	dsRNA present (3/3)	Amplicon present	Amplicon present	Hypovirulent
162	CP21-06	Intermediate	CPi9-01	dsRNA present (3/3)	Amplicon present	Amplicon present	Hypovirulent
163	CP21-07	Intermediate	CPi9-19	dsRNA absent (0/3)	-	-	Virulent
164	CP21-08	Intermediate	CPi10-40	dsRNA absent (0/3)	-	-	Virulent
165	CP21-09	Intermediate	CPi10-37	dsRNA absent (0/3)	-	-	Virulent
166	CP22-01	Intermediate	CPi7-13	dsRNA absent (0/3)	-	-	Virulent
167	CP22-02	Intermediate	CPi11-21	dsRNA absent (0/3)	-	-	Virulent
168	CP22-03	Intermediate	CPi15-07	dsRNA absent (0/3)	-	-	Virulent
169	CP22-04	Virulent	CPi12-23	dsRNA absent (0/3)	-	-	Virulent
170	CP22-05	Virulent	CPi11-36	dsRNA absent (0/3)	-	-	Virulent
171	CP22-06	Intermediate	CPi12-06	dsRNA absent (0/3)	-	-	Virulent
172	CP22-07	Intermediate	CPi12-27	dsRNA absent (0/3)	-	-	Virulent
173	CP22-08	Hypovirulent	CPi13-03	dsRNA present (3/3)	Amplicon present	Amplicon present	Hypovirulent
174	CP22-09	Intermediate	CPi12-11	dsRNA absent (0/3)	-	-	Virulent
175	CP22-10	Hypovirulent	CPi8-17	dsRNA absent (0/3)	-	-	Virulent
176	CP23-01	Virulent	CPi8-12	dsRNA absent (0/3)	-	-	Virulent
177	CP23-02	Hypovirulent	CPi8-11	dsRNA present (3/3)	Amplicon present	Amplicon present	Hypovirulent
178	CP23-03	Intermediate	CPi10-22	dsRNA absent (0/3)	-	-	Virulent
179	CP23-04	Intermediate	CPi7-20	dsRNA absent (0/3)	-	-	Virulent

No.	Isolate code	Phenotypic assessment	Sample code immunoassay	Result immunoassay*	Result RT-PCR ORF-A	Result RT-PCR ORF-B	Classification of isolate
180	CP23-05	Intermediate	CPi11-18	dsRNA absent (0/3)	No amplicon	No amplicon	Virulent
181	CP23-06	Intermediate	CPi12-21	dsRNA absent (0/3)	No amplicon	No amplicon	Virulent
182	CP23-07	Virulent	CPi15-06	dsRNA absent (0/3)	-	-	Virulent
183	CP23-08	Virulent	CPi15-27	dsRNA absent (0/3)	-	-	Virulent
184	CP23-09	Intermediate	CPi10-33	dsRNA absent (0/3)	-	-	Virulent
185	CP23-10	Intermediate	CPi11-35	dsRNA absent (0/3)	Amplicon present	Amplicon present	Hypovirulent
186	CP23-11	Intermediate	CPi10-23	dsRNA absent (0/3)	-	-	Virulent
187	CP23-12	Intermediate	CPi10-21	dsRNA absent (0/3)	-	-	Virulent
188	CP23-13	Intermediate	CPi8-09	dsRNA present (1/3)	Amplicon present	Amplicon present	Hypovirulent
189	CP23-14	Intermediate	CPi11-33	dsRNA absent (0/3)	No amplicon	No amplicon	Virulent
190	CP23-15	Intermediate	CPi12-24	dsRNA absent (0/3)	-	-	Virulent
191	CP23-16	Intermediate	CPi9-17	dsRNA absent (0/3)	-	-	Virulent
192	CP24-01	Virulent	CPi12-22	dsRNA absent (0/3)	No amplicon	No amplicon	Virulent
193	CP24-02	Intermediate	CPi11-24	dsRNA absent (0/3)	No amplicon	No amplicon	Virulent
194	CP24-03	Intermediate	CPi15-09	dsRNA absent (0/3)	-	-	Virulent
195	CP24-04	Intermediate	CPi11-20	dsRNA absent (0/3)	-	-	Virulent
196	CP24-05	Intermediate	CPi11-19	dsRNA absent (0/3)	-	-	Virulent
197	CP24-06	Virulent	CPi11-15	dsRNA absent (0/3)	-	-	Virulent
198	CP24-07	Intermediate	CPi8-13	dsRNA absent (0/3)	-	-	Virulent
199	CP24-08	Intermediate	CPi9-26	dsRNA absent (0/3)	-	-	Virulent
200	CP24-09	Intermediate	CPi11-04	dsRNA present(3/3)	Amplicon present	Amplicon present	Hypovirulent
201	CP24-10	Intermediate	CPi15-10	dsRNA absent (0/3)	-	-	Virulent
202	CP24-11	Intermediate	CPi10-28	dsRNA absent (0/3)	-	-	Virulent
203	CP24-12	Intermediate	CPi15-08	dsRNA absent (0/3)	-	-	Virulent
204	CP24-13	Intermediate	CPi12-01	dsRNA absent (0/3)	-	-	Virulent
205	CP24-14	Virulent	CPi10-32	dsRNA absent (0/3)	-	-	Virulent
206	CP24-15	Intermediate	CPi8-02	dsRNA absent (0/3)	-	-	Virulent
207	CP24-16	Hypovirulent	CPi12-47	dsRNA present (3/3)	Amplicon present	Amplicon present	Hypovirulent
208	CP24-17	Virulent	CPi11-26	dsRNA absent (0/3)	No amplicon	No amplicon	Virulent
209	CP24-18	Intermediate	CPi10-07	dsRNA absent (0/3)	-	-	Virulent
210	CP24-19	Hypovirulent	CPi11-16	dsRNA present (1/3)	Amplicon present	Amplicon present	Hypovirulent
211	CP24-20	Intermediate	CPi9-22	dsRNA absent (0/3)	-	-	Virulent
212	CP25-01	Virulent	CPi7-17	dsRNA absent (0/3)	-	-	Virulent
213	CP25-02	Intermediate	CPi9-13	dsRNA absent (0/3)	-	-	Virulent
214	CP25-03	Hypovirulent	CPi7-06	dsRNA present (3/3)	Amplicon present	Amplicon present	Hypovirulent
215	CP25-04	Intermediate	CPi12-36	dsRNA absent (0/3)	-	-	Virulent
216	CP25-05	Hypovirulent	CPi7-09	dsRNA present (2/3)	Amplicon present	Amplicon present	Hypovirulent
217	CP25-06	Hypovirulent	CPi8-07	dsRNA absent (0/3)	-	-	Virulent
218	CP25-07	Intermediate	CPi7-02	dsRNA present (3/3)	Amplicon present	Amplicon present	Hypovirulent
219	CP25-08	Hypovirulent	CPi10-15	dsRNA absent (0/3)	-	-	Virulent
220	CP25-09	Intermediate	CPi7-10	dsRNA absent (0/3)	No amplicon	No amplicon	Virulent
221	CP25-10	Intermediate	CPi7-12	dsRNA present (3/3)	Amplicon present	Amplicon present	Hypovirulent
222	CP25-11	Hypovirulent	CPi8-06	dsRNA absent (0/3)	-	-	Virulent
223	CP25-12	Intermediate	CPi12-41	dsRNA absent (0/3)	-	-	Virulent
224	CP25-13	Intermediate	CPi9-21	dsRNA absent (0/3)	-	-	Virulent
225	CP25-14	Intermediate	CPi11-10	dsRNA absent (0/3)	-	-	Virulent
226	CP25-15	Intermediate	CPi9-28	dsRNA absent (0/3)	-	-	Virulent
227	CP25-16	Intermediate	CPi9-12	dsRNA present (3/3)	Amplicon present	Amplicon present	Hypovirulent
228	CP25-17	Intermediate	CPi11-17	dsRNA absent (0/3)	-	-	Virulent
229	CP25-18	Virulent	CPi7-16	dsRNA absent (0/3)	-	-	Virulent
230	CP25-19	Intermediate	CPi11-30	dsRNA absent (0/3)	-	-	Virulent
231	CP25-20	Intermediate	CPi11-13	dsRNA absent (0/3)	-	-	Virulent
232	CP25-21	Intermediate	CPi8-08	dsRNA absent (0/3)	-	-	Virulent
233	CP25-22	Intermediate	CPi12-40	dsRNA present (1/3)	Amplicon present	Amplicon present	Hypovirulent
234	CP25-23	Intermediate	CPi11-11	dsRNA absent (0/3)	-	-	Virulent
235	CP25-24	Intermediate	CPi9-09	dsRNA absent (0/3)	-	-	Virulent
236	CP25-25	Intermediate	CPi9-27	dsRNA absent (0/3)	-	-	Virulent
237	CP25-26	Intermediate	CPi9-16	dsRNA absent (0/3)	-	-	Virulent
238	CP25-27	Intermediate	CPi12-07	dsRNA absent (0/3)	No amplicon	No amplicon	Virulent
239	CP26-01	Hypovirulent	CPi15-25	dsRNA absent (0/3)	-	-	Virulent
240	CP26-03	Hypovirulent	CPi10-03	dsRNA present (3/3)	Amplicon present	Amplicon present	Hypovirulent

No.	Isolate code	Phenotypic assessment	Sample code immunoassay	Result immunoassay*	Result RT-PCR ORF-A	Result RT-PCR ORF-B	Classification of isolate
241	CP26-05	Hypovirulent	CPi7-23	dsRNA present (3/3)	Amplicon present	Amplicon present	Hypovirulent
242	CP26-06	Hypovirulent	CPi8-21	dsRNA present (3/3)	Amplicon present	Amplicon present	Hypovirulent
243	CP26-07	Hypovirulent	CPi7-01	dsRNA present (3/3)	Amplicon present	Amplicon present	Hypovirulent
244	CP26-08	Hypovirulent	CPi11-01	dsRNA present (3/3)	Amplicon present	Amplicon present	Hypovirulent
245	CP26-09	Hypovirulent	CPi12-31	dsRNA present (3/3)	No amplicon	No amplicon	Virulent
246	CP26-10	Intermediate	CPi10-20	dsRNA absent (0/3)	-	-	Virulent
247	CP26-11	Hypovirulent	CPi15-04	dsRNA present (1/3)	No amplicon	No amplicon	Virulent
248	CP26-12	Intermediate	CPi10-26	dsRNA absent (0/3)	-	-	Virulent
249	CP26-13	Hypovirulent	CPi12-39	dsRNA present (3/3)	Amplicon present	Amplicon present	Hypovirulent
250	CP26-14	Intermediate	CPi12-17	dsRNA absent (0/3)	-	-	Virulent
251	CP26-15	Intermediate	CPi12-19	dsRNA absent (0/3)	-	-	Virulent
252	CP26-16	Hypovirulent	CPi9-04	dsRNA present (3/3)	Amplicon present	Amplicon present	Hypovirulent
253	CP26-17	Hypovirulent	CPi10-41	dsRNA present (3/3)	Amplicon present	Amplicon present	Hypovirulent
254	CP26-18	Virulent	CPi12-10	dsRNA absent (0/3)	-	-	Virulent
255	CP26-19	Intermediate	CPi11-34	dsRNA absent (0/3)	-	-	Virulent
256	CP26-20	Intermediate	CPi11-31	dsRNA absent (0/3)	-	-	Virulent
257	CP27-02	Intermediate	CPi11-37	dsRNA present (1/3)	Amplicon present	Amplicon present	Hypovirulent
258	CP27-03	Intermediate	CPi15-05	dsRNA present (1/3)	Amplicon present	No amplicon	Hypovirulent
259	CP27-04	Intermediate	CPi12-12	dsRNA absent (0/3)	-	-	Virulent
260	CP27-05	Intermediate	CPi11-28	dsRNA absent (0/3)	-	-	Virulent
261	CP27-06	Intermediate	CPi12-48	dsRNA absent (0/3)	-	-	Virulent
262	CP27-08	Intermediate	CPi10-35	dsRNA absent (0/3)	-	-	Virulent
263	CP27-12	Intermediate	CPi9-25	dsRNA present (2/3)	No amplicon	No amplicon	Virulent

* Numbers in parentheses separated by slashes indicate how many of the triplicate reactions resulted in a visible dot on the membrane indicating the presence of dsRNA; the minus sign (-) indicates samples that were not analyzed by RT-PCR

Supplementary Table 4. Haplotypes of CHV-1 based on the sequencing of a 1,202 bp fragment of ORF-A found in South Tyrol, Italy. GenBank accession numbers of each haplotype and the location of each haplotype are also included.

Haplotype	GenBank accession No.	Number of sequences	Isolates with the haplotype	Sample code	Farm	District
HA1	ON022074	10	CP06-03	WIN-8	WIN	Vinschgau – Val Venosta
			CP15-14	NOP-6	NOP	Salten-Schlern – Salto-Sciliar
			CP15-13	NOP-3	NOP	Salten-Schlern – Salto-Sciliar
			CP02-11	NOP-5	NOP	Salten-Schlern – Salto-Sciliar
			CP14-08	MAR-8	MAR	Salten-Schlern – Salto-Sciliar
			CP02-15	NOP-5	NOP	Salten-Schlern – Salto-Sciliar
			CP12-01	WIB-8	WIB	Burggrafenamt – Burgraviato
			CP26-13	MONT-20	MONT	Monticolo Forest
			CP10-18	NOP-3	NOP	Salten-Schlern – Salto-Sciliar
			CP10-19	NOC-3	NOC	Salten-Schlern – Salto-Sciliar
HA2	ON022075	1	CP11-06	JOG-6	JOG	Salten-Schlern – Salto-Sciliar
HA3	ON022076	3	CP24-16	MONT-31	MONT	Monticolo Forest
			CP10-09	UNT-9	UNT	Überetsch-Unterland – Oltradige-Bassa Atesina
			CP14-06	WIN-2	WIN	Vinschgau – Val Venosta
HA4	ON022077	1	CP09-09	VET-5	VET	Vinschgau – Val Venosta
HA5	ON022078	2	CP25-10	HOAN-7	HOAN	Überetsch-Unterland – Oltradige-Bassa Atesina
			CP15-02	GOG-12	GOG	Vinschgau – Val Venosta
HA6	ON022079	1	CP15-15	OBK-8	OBK	Eisacktal – Valle Isarco
HA7	ON022080	1	CP24-09	MONT-12	MONT	Monticolo Forest
HA8	ON022081	2	CP09-18	OBG-9	OBG	Eisacktal – Valle Isarco
			CP21-02	PLA-6	PLA	Salten-Schlern – Salto-Sciliar
HA9	ON022082	1	CP23-02	BRUN-4	BRUN	Burggrafenamt – Burgraviato
HA10	ON022083	1	CP26-08	CRIS-6	CRIS	Burggrafenamt – Burgraviato
HA11	ON022084	1	CP26-03	ALAL-5	ALAL	Burggrafenamt – Burgraviato
HA12	ON022085	7	CP14-17	AUF-9	AUF	Vinschgau – Val Venosta
			CP14-10	UNT-5	UNT	Überetsch-Unterland – Oltradige-Bassa Atesina
			CP02-16	NOP-6	NOP	Salten-Schlern – Salto-Sciliar
			CP22-08	RUTT-4	RUTT	Überetsch-Unterland – Oltradige-Bassa Atesina
			CP20-01	UNT-12	UNT	Überetsch-Unterland – Oltradige-Bassa Atesina
			CP02-06	UNT-4	UNT	Überetsch-Unterland – Oltradige-Bassa Atesina
			CP11-18	GOG-7	GOG	Vinschgau – Val Venosta
HA13	ON022086	1	CP23-10	WEIR-4	WEIR	Vinschgau – Val Venosta
HA14	ON022087	1	CP19-03	PLA-2	PLA	Salten-Schlern – Salto-Sciliar
HA15	ON022088	2	CP21-05	AUF-11	AUF	Vinschgau – Val Venosta
			CP21-06	AUF-12	AUF	Vinschgau – Val Venosta
HA16	ON022089	1	CP16-03	TAP-1	TAP	Vinschgau – Val Venosta
HA17	ON022090	1	CP09-16	PLA-3	PLA	Salten-Schlern – Salto-Sciliar
HA18	ON022091	4	CP15-06	TES-4	TES	Salten-Schlern – Salto-Sciliar
			CP16-02	ISC-1	ISC	Eisacktal – Valle Isarco
			CP10-16	VET-9	VET	Vinschgau – Val Venosta
			CP02-17	TAP-8	TAP	Vinschgau – Val Venosta
HA19	ON022092	1	CP20-03	UNT-11	UNT	Überetsch-Unterland – Oltradige-Bassa Atesina
HA20	ON022093	1	CP26-16	RUTT-8	RUTT	Überetsch-Unterland – Oltradige-Bassa Atesina
HA21	ON022094	1	CP02-09	ISC-10	ISC	Eisacktal – Valle Isarco
HA22	ON022095	1	CP26-05	MOAR-9	MOAR	Burggrafenamt – Burgraviato
HA23	ON022096	2	CP25-07	MOAR-12	MOAR	Burggrafenamt – Burgraviato
			CP26-06	MOAR-12	MOAR	Burggrafenamt – Burgraviato

Supplementary Table 5. Haplotypes of CHV-1 based on the sequencing of a 1,194 bp fragment of ORF-B found in South Tyrol, Italy. GenBank accession numbers of each haplotype and the location of each haplotype are also included.

Haplotype	GenBank accession No.	Number of sequences	Isolates with the haplotype	Sample code	Farm	District
HB1	ON022044	1	CP25-16	BLAS-4	BLAS	Burggrafenamt – Burgraviato
HB2	ON022045	2	CP26-07	CRIS-3	CRIS	Burggrafenamt – Burgraviato
			CP26-08	CRIS-6	CRIS	Burggrafenamt – Burgraviato
HB3	ON022046	1	CP26-16	RUTT-8	RUTT	Überetsch-Unterland – Oltradige-Bassa Atesina
HB4	ON022047	3	CP25-07	MOAR-12	MOAR	Burggrafenamt – Burgraviato
			CP26-05	MOAR-9	MOAR	Burggrafenamt – Burgraviato
			CP26-06	MOAR-12	MOAR	Burggrafenamt – Burgraviato
HB5	ON022048	1	CP11-18	GOG-7	GOG	Vinschgau – Val Venosta
HB6	ON022049	1	CP24-09	MONT-12	MONT	Monticolo Forest
HB7	ON022050	1	CP23-10	WEIR-4	WEIR	Vinschgau – Val Venosta
HB8	ON022051	2	CP21-05	AUF-11	AUF	Vinschgau – Val Venosta
			CP21-06	AUF-12	AUF	Vinschgau – Val Venosta
HB9	ON022052	5	CP14-09	UNT-3	UNT	Überetsch-Unterland – Oltradige-Bassa Atesina
			CP14-10	UNT-5	UNT	Überetsch-Unterland – Oltradige-Bassa Atesina
			CP14-17	AUF-9	AUF	Vinschgau – Val Venosta
			CP20-01	UNT-12	UNT	Überetsch-Unterland – Oltradige-Bassa Atesina
			CP22-08	RUTT-4	RUTT	Überetsch-Unterland – Oltradige-Bassa Atesina
HB10	ON022053	2	CP02-06	UNT-4	UNT	Überetsch-Unterland – Oltradige-Bassa Atesina
			CP02-16	NOP-6	NOP	Salten-Schlern – Salto-Sciliar
HB11	ON022054	1	CP26-03	ALAL-5	ALAL	Burggrafenamt – Burgraviato
HB12	ON022055	5	CP02-09	ISC-10	ISC	Eisacktal – Valle Isarco
			CP02-17	TAP-8	TAP	Vinschgau – Val Venosta
			CP15-06	TES-4	TES	Salten-Schlern – Salto-Sciliar
			CP20-12	ISC-3	ISC	Eisacktal – Valle Isarco
			CP16-02	ISC-1	ISC	Eisacktal – Valle Isarco
HB13	ON022056	1	CP15-02	GOG-12	GOG	Vinschgau – Val Venosta
HB14	ON022057	2	CP09-18	OBG-9	OBG	Eisacktal – Valle Isarco
			CP21-02	PLA-6	PLA	Salten-Schlern – Salto-Sciliar
HB15	ON022058	1	CP10-16	VET-9	VET	Vinschgau – Val Venosta
HB16	ON022059	1	CP15-07	NOP-8	NOP	Salten-Schlern – Salto-Sciliar
HB17	ON022060	7	CP10-19	NOC-3	NOC	Salten-Schlern – Salto-Sciliar
			CP02-11	NOP-5	NOP	Salten-Schlern – Salto-Sciliar
			CP02-15	NOP-5	NOP	Salten-Schlern – Salto-Sciliar
			CP15-13	NOP-3	NOP	Salten-Schlern – Salto-Sciliar
			CP21-01	PLA-5	PLA	Salten-Schlern – Salto-Sciliar
			CP26-13	MONT-20	MONT	Monticolo Forest
			CP10-18	NOP-3	NOP	Salten-Schlern – Salto-Sciliar
HB18	ON022061	5	CP09-15	GOG-2	GOG	Vinschgau – Val Venosta
			CP11-06	JOG-6	JOG	Salten-Schlern – Salto-Sciliar
			CP14-06	WIN-2	WIN	Vinschgau – Val Venosta
			CP24-16	MONT-31	MONT	Monticolo Forest
			CP10-09	UNT-9	UNT	Überetsch-Unterland – Oltradige-Bassa Atesina
HB19	ON022062	1	CP09-09	VET-5	VET	Vinschgau – Val Venosta
HB20	ON022063	2	CP12-10	KGK-1	KGK	Vinschgau – Val Venosta
			CP12-18	FLK-4	FLK	Salten-Schlern – Salto-Sciliar
HB21	ON022064	1	CP14-18	SPR-10	SPR	Eisacktal – Valle Isarco
HB22	ON022065	1	CP20-10	JOG-9	JOG	Salten-Schlern – Salto-Sciliar
HB23	ON022066	1	CP27-02	BRUN-6	BRUN	Burggrafenamt – Burgraviato
HB24	ON022067	1	CP19-03	PLA-2	PLA	Salten-Schlern – Salto-Sciliar
HB25	ON022068	1	CP26-17	HOAN-10	HOAN	Überetsch-Unterland – Oltradige-Bassa Atesina
HB26	ON022069	1	CP16-03	TAP-1	TAP	Vinschgau – Val Venosta
HB27	ON022070	1	CP20-03	UNT-11	UNT	Überetsch-Unterland – Oltradige-Bassa Atesina

Haplotype	GenBank accession No.	Number of sequences	Isolates with the haplotype	Sample code	Farm	District
HB28	ON022071	1	CP25-03	MOAR-7	MOAR	Burggrafenamt – Burgraviato
HB29	ON022072	1	CP25-10	HOAN-7	HOAN	Überetsch-Unterland – Oltradige-Bassa Atesina
HB30	ON022073	2	CP23-13	RUTT-4	RUTT	Überetsch-Unterland – Oltradige-Bassa Atesina
			CP25-05	MOAR-8	MOAR	Burggrafenamt – Burgraviato

Supplementary Table 6. Haplotypes of CHV-1 based on the combined sequences of fragments of ORF-A and ORF-B found in South Tyrol. The location of each combined haplotype is also included in the table.

Haplotypes	Number of sequences	Isolates with the haplotype	Sample code	Farm	District
HAB1	2	CP02-06	UNT-4	UNT	Überetsch-Unterland – Oltradige-Bassa Atesina
		CP02-16	NOP-6	NOP	Salten-Schlern – Salto-Sciliar
HAB2	1	CP02-09	ISC-10	ISC	Eisacktal – Valle Isarco
HAB3	6	CP02-11	NOP-5	NOP	Salten-Schlern – Salto-Sciliar
		CP02-15	NOP-5	NOP	Salten-Schlern – Salto-Sciliar
		CP10-18	NOP-3	NOP	Salten-Schlern – Salto-Sciliar
		CP10-19	NOC-3	NOC	Salten-Schlern – Salto-Sciliar
		CP15-13	NOP-3	NOP	Salten-Schlern – Salto-Sciliar
		CP26-13	MONT-20	MONT	Monticolo Forest
HAB4	3	CP02-17	TAP-8	TAP	Vinschgau – Val Venosta
		CP15-06	TES-4	TES	Salten-Schlern – Salto-Sciliar
		CP16-02	ISC-1	ISC	Eisacktal – Valle Isarco
HAB5	1	CP09-09	VET-5	VET	Vinschgau – Val Venosta
HAB6	2	CP09-18	OBG-9	OBG	Eisacktal – Valle Isarco
		CP21-02	PLA-6	PLA	Salten-Schlern – Salto-Sciliar
HAB7	3	CP10-09	UNT-9	UNT	Überetsch-Unterland – Oltradige-Bassa Atesina
		CP14-06	WIN-2	WIN	Vinschgau – Val Venosta
		CP24-16	MONT-31	MONT	Monticolo Forest
HAB8	1	CP10-16	VET-9	VET	Vinschgau – Val Venosta
HAB9	1	CP11-06	JOG-6	JOG	Salten-Schlern – Salto-Sciliar
HAB10	1	CP11-18	GOG-7	GOG	Vinschgau – Val Venosta
HAB11	4	CP14-10	UNT-5	UNT	Überetsch-Unterland – Oltradige-Bassa Atesina
		CP14-17	AUF-9	AUF	Vinschgau – Val Venosta
		CP20-01	UNT-12	UNT	Überetsch-Unterland – Oltradige-Bassa Atesina
		CP22-08	RUTT-4	RUTT	Überetsch-Unterland – Oltradige-Bassa Atesina
HAB12	1	CP15-02	GOG-12	GOG	Vinschgau – Val Venosta
HAB13	1	CP16-03	TAP-1	TAP	Vinschgau – Val Venosta
HAB14	1	CP19-03	PLA-2	PLA	Salten-Schlern – Salto-Sciliar
HAB15	1	CP20-03	UNT-11	UNT	Überetsch-Unterland – Oltradige-Bassa Atesina
HAB16	2	CP21-05	AUF-11	AUF	Vinschgau – Val Venosta
		CP21-06	AUF-12	AUF	Vinschgau – Val Venosta
HAB17	1	CP23-10	WEIR-4	WEIR	Vinschgau – Val Venosta
HAB18	1	CP24-09	MONT-12	MONT	Monticolo Forest
HAB19	2	CP25-07	MOAR-12	MOAR	Burggrafenamt – Burgraviato
		CP26-06	MOAR-12	MOAR	Burggrafenamt – Burgraviato
HAB20	1	CP25-10	HOAN-7	HOAN	Überetsch-Unterland – Oltradige-Bassa Atesina
HAB21	1	CP26-03	ALAL-5	ALAL	Burggrafenamt – Burgraviato
HAB22	1	CP26-05	MOAR-9	MOAR	Burggrafenamt – Burgraviato
HAB23	1	CP26-08	CRIS-6	CRIS	Burggrafenamt – Burgraviato
HAB24	1	CP26-16	RUTT-8	RUTT	Überetsch-Unterland – Oltradige-Bassa Atesina

Supplementary Table 7. K_a/K_s values of different haplotype combinations of CHV-1 based on ORF-A, calculated in TBtools (Chen et al., 2020).

Haplotype pair	K_a	K_s	K_a/K_s
HA2 - HA1	0.003382	0.013101	0.258167
HA3 - HA1	0.006762	0.013072	0.517271
HA4 - HA1	0.012427	0.033246	0.373794
HA5 - HA1	0.012427	0.040076	0.310091
HA6 - HA1	0.013565	0.040099	0.338286
HA7 - HA1	0.013611	0.019781	0.688070
HA8 - HA1	0.013571	0.029830	0.454952
HA9 - HA1	0.015848	0.023136	0.685001
HA10 - HA1	0.013574	0.026442	0.513346
HA11 - HA1	0.005626	0.029805	0.188773
HA12 - HA1	0.012447	0.023009	0.540968
HA13 - HA1	0.014722	0.019722	0.746466
HA14 - HA1	0.011302	0.023041	0.490512
HA15 - HA1	0.013569	0.023110	0.587120
HA16 - HA1	0.010144	0.026544	0.382151
HA17 - HA1	0.010164	0.029756	0.341575
HA18 - HA1	0.010164	0.033136	0.306731
HA19 - HA1	0.011293	0.026449	0.426982
HA20 - HA1	0.012430	0.023104	0.537984
HA21 - HA1	0.010188	0.019765	0.515448
HA22 - HA1	0.009026	0.019716	0.457795
HA23 - HA1	0.009029	0.019695	0.458469
HA1 - HA2	0.003382	0.013101	0.258167
HA3 - HA2	0.002254	0.000000	0.000000
HA4 - HA2	0.010183	0.040121	0.253809
HA5 - HA2	0.010183	0.047022	0.216562
HA6 - HA2	0.011321	0.047048	0.240626
HA7 - HA2	0.011360	0.026522	0.428305
HA8 - HA2	0.011326	0.036664	0.308924
HA9 - HA2	0.013605	0.029913	0.454810
HA10 - HA2	0.011328	0.033237	0.340836
HA11 - HA2	0.005643	0.036633	0.154050
HA12 - HA2	0.010199	0.029748	0.342863
HA13 - HA2	0.012475	0.026442	0.471810
HA14 - HA2	0.007913	0.029838	0.265186
HA15 - HA2	0.011324	0.029879	0.378995
HA16 - HA2	0.007901	0.033367	0.236808
HA17 - HA2	0.009050	0.036633	0.247040
HA18 - HA2	0.009050	0.040054	0.225942
HA19 - HA2	0.009048	0.033246	0.272155
HA20 - HA2	0.010185	0.029871	0.340964
HA21 - HA2	0.007936	0.026500	0.299464
HA22 - HA2	0.006780	0.026434	0.256472
HA23 - HA2	0.006782	0.026405	0.256852
HA1 - HA3	0.006762	0.013072	0.517271
HA2 - HA3	0.002254	0.000000	0.000000
HA4 - HA3	0.012425	0.040099	0.309860
HA5 - HA3	0.012425	0.046995	0.264388
HA6 - HA3	0.013562	0.047022	0.288426
HA7 - HA3	0.013608	0.026507	0.513380
HA8 - HA3	0.013569	0.036643	0.370288

Haplotype pair	K_a	K_s	K_a/K_s
HA9 - HA3	0.013561	0.029896	0.453605
HA10 - HA3	0.011292	0.033219	0.339934
HA11 - HA3	0.007887	0.036613	0.215427
HA12 - HA3	0.012445	0.029731	0.418578
HA13 - HA3	0.014719	0.026427	0.556959
HA14 - HA3	0.011300	0.029772	0.379535
HA15 - HA3	0.011288	0.029863	0.377992
HA16 - HA3	0.007876	0.033348	0.236185
HA17 - HA3	0.012439	0.036552	0.340310
HA18 - HA3	0.012439	0.039965	0.311248
HA19 - HA3	0.011291	0.033228	0.339808
HA20 - HA3	0.012427	0.029855	0.416260
HA21 - HA3	0.009046	0.026500	0.341342
HA22 - HA3	0.009024	0.026420	0.341574
HA23 - HA3	0.009028	0.026391	0.342079
HA1 - HA4	0.012427	0.033246	0.373794
HA2 - HA4	0.010183	0.040121	0.253809
HA3 - HA4	0.012425	0.040099	0.309860
HA5 - HA4	0.011272	0.040256	0.280012
HA6 - HA4	0.012406	0.040278	0.308016
HA7 - HA4	0.013596	0.019847	0.685024
HA8 - HA4	0.012412	0.029962	0.414257
HA9 - HA4	0.014683	0.023238	0.631835
HA10 - HA4	0.012414	0.026559	0.467433
HA11 - HA4	0.009009	0.029937	0.300931
HA12 - HA4	0.011290	0.023110	0.488526
HA13 - HA4	0.013558	0.019809	0.684472
HA14 - HA4	0.010149	0.023142	0.438531
HA15 - HA4	0.012410	0.023213	0.534613
HA16 - HA4	0.008996	0.026662	0.337425
HA17 - HA4	0.011285	0.029888	0.377572
HA18 - HA4	0.011285	0.026507	0.425721
HA19 - HA4	0.010141	0.026566	0.381726
HA20 - HA4	0.011274	0.023206	0.485824
HA21 - HA4	0.009035	0.013176	0.685732
HA22 - HA4	0.007880	0.019803	0.397917
HA23 - HA4	0.010151	0.019781	0.513136
HA1 - HA5	0.012427	0.040076	0.310091
HA2 - HA5	0.010183	0.047022	0.216562
HA3 - HA5	0.012425	0.046995	0.264388
HA4 - HA5	0.011272	0.040256	0.280012
HA6 - HA5	0.001119	0.000000	0.000000
HA7 - HA5	0.011312	0.026581	0.425586
HA8 - HA5	0.003365	0.023200	0.145036
HA9 - HA5	0.012405	0.026618	0.466048
HA10 - HA5	0.010142	0.033348	0.304124
HA11 - HA5	0.009009	0.036756	0.245108
HA12 - HA5	0.009018	0.029846	0.302161
HA13 - HA5	0.011282	0.026529	0.425248
HA14 - HA5	0.010149	0.029888	0.339558
HA15 - HA5	0.010138	0.026588	0.381302
HA16 - HA5	0.008996	0.030062	0.299259
HA17 - HA5	0.009014	0.036694	0.245656
HA18 - HA5	0.009014	0.040121	0.224675
HA19 - HA5	0.007876	0.033357	0.236097
HA20 - HA5	0.009006	0.029971	0.300485
HA21 - HA5	0.006766	0.026588	0.254486

Haplotype pair	K_a	K_s	K_a/K_s
HA22 - HA5	0.005620	0.026522	0.211903
HA23 - HA5	0.007883	0.026493	0.297551
HA1 - HA6	0.013565	0.040099	0.338286
HA2 - HA6	0.011321	0.047048	0.240626
HA3 - HA6	0.013562	0.047022	0.288426
HA4 - HA6	0.012406	0.040278	0.308016
HA5 - HA6	0.001119	0.000000	0.000000
HA7 - HA6	0.012451	0.026596	0.468152
HA8 - HA6	0.004489	0.023213	0.193383
HA9 - HA6	0.011267	0.026632	0.423046
HA10 - HA6	0.009007	0.033367	0.269928
HA11 - HA6	0.010141	0.036776	0.275749
HA12 - HA6	0.010151	0.029863	0.339936
HA13 - HA6	0.012417	0.026544	0.467780
HA14 - HA6	0.011283	0.029904	0.377292
HA15 - HA6	0.011271	0.026603	0.423674
HA16 - HA6	0.010127	0.030079	0.336669
HA17 - HA6	0.010147	0.036715	0.276366
HA18 - HA6	0.010147	0.040143	0.252762
HA19 - HA6	0.009006	0.033376	0.269827
HA20 - HA6	0.010137	0.029987	0.338049
HA21 - HA6	0.007899	0.026603	0.296904
HA22 - HA6	0.006748	0.026537	0.254286
HA23 - HA6	0.009014	0.026507	0.340063
HA1 - HA7	0.013611	0.019781	0.688070
HA2 - HA7	0.011360	0.026522	0.428305
HA3 - HA7	0.013608	0.026507	0.513380
HA4 - HA7	0.013596	0.019847	0.685024
HA5 - HA7	0.011312	0.026581	0.425586
HA6 - HA7	0.012451	0.026596	0.468152
HA8 - HA7	0.012457	0.016489	0.755465
HA9 - HA7	0.012450	0.009866	1.261886
HA10 - HA7	0.010178	0.013155	0.773741
HA11 - HA7	0.010175	0.016493	0.616943
HA12 - HA7	0.006778	0.009812	0.690760
HA13 - HA7	0.009044	0.006541	1.382580
HA14 - HA7	0.012467	0.009825	1.268869
HA15 - HA7	0.010174	0.009855	1.032408
HA16 - HA7	0.011303	0.013205	0.855933
HA17 - HA7	0.011325	0.016448	0.688547
HA18 - HA7	0.011325	0.019781	0.572521
HA19 - HA7	0.010177	0.013158	0.773456
HA20 - HA7	0.009038	0.009852	0.917339
HA21 - HA7	0.006791	0.006556	1.035839
HA22 - HA7	0.007908	0.006540	1.209286
HA23 - HA7	0.009045	0.006540	1.383087
HA1 - HA8	0.013571	0.029830	0.454952
HA2 - HA8	0.011326	0.036664	0.308924
HA3 - HA8	0.013569	0.036643	0.370288
HA4 - HA8	0.012412	0.029962	0.414257
HA5 - HA8	0.003365	0.023200	0.145036
HA6 - HA8	0.004489	0.023213	0.193383
HA7 - HA8	0.012457	0.016489	0.755465
HA9 - HA8	0.013547	0.019858	0.682199
HA10 - HA8	0.011281	0.023168	0.486904

Haplotype pair	K_a	K_s	K_a/K_s
HA11 - HA8	0.010146	0.026529	0.382434
HA12 - HA8	0.010156	0.019749	0.514272
HA13 - HA8	0.011285	0.016457	0.685710
HA14 - HA8	0.011288	0.019776	0.570793
HA15 - HA8	0.011276	0.019836	0.568480
HA16 - HA8	0.010131	0.023258	0.435619
HA17 - HA8	0.010151	0.026486	0.383284
HA18 - HA8	0.010151	0.029863	0.339936
HA19 - HA8	0.009010	0.023174	0.388792
HA20 - HA8	0.010142	0.019830	0.511434
HA21 - HA8	0.007902	0.016493	0.479120
HA22 - HA8	0.006751	0.016453	0.410338
HA23 - HA8	0.009018	0.016435	0.548749
HA1 - HA9	0.015848	0.023136	0.685001
HA2 - HA9	0.013605	0.029913	0.454810
HA3 - HA9	0.013561	0.029896	0.453605
HA4 - HA9	0.014683	0.023238	0.631835
HA5 - HA9	0.012405	0.026618	0.466048
HA6 - HA9	0.011267	0.026632	0.423046
HA7 - HA9	0.012450	0.009866	1.261886
HA8 - HA9	0.013547	0.019858	0.682199
HA10 - HA9	0.006744	0.009858	0.684146
HA11 - HA9	0.012412	0.019841	0.625569
HA12 - HA9	0.010151	0.013129	0.773112
HA13 - HA9	0.012416	0.009847	1.260858
HA14 - HA9	0.013558	0.013147	1.031261
HA15 - HA9	0.006742	0.013187	0.511226
HA16 - HA9	0.007864	0.016566	0.474690
HA17 - HA9	0.012419	0.019809	0.626958
HA18 - HA9	0.012419	0.023161	0.536198
HA19 - HA9	0.010705	0.018178	0.588931
HA20 - HA9	0.010136	0.013184	0.768856
HA21 - HA9	0.007898	0.009869	0.800297
HA22 - HA9	0.009010	0.009844	0.915249
HA23 - HA9	0.011284	0.009834	1.147474
HA1 - HA10	0.013574	0.026442	0.513346
HA2 - HA10	0.011328	0.033237	0.340836
HA3 - HA10	0.011292	0.033219	0.339934
HA4 - HA10	0.012414	0.026559	0.467433
HA5 - HA10	0.010142	0.033348	0.304124
HA6 - HA10	0.009007	0.033367	0.269928
HA7 - HA10	0.010178	0.013155	0.773741
HA8 - HA10	0.011281	0.023168	0.486904
HA9 - HA10	0.006744	0.009858	0.684146
HA11 - HA10	0.010148	0.023149	0.438369
HA12 - HA10	0.007889	0.016412	0.480676
HA13 - HA10	0.010151	0.013129	0.773112
HA14 - HA10	0.011290	0.016435	0.686974
HA15 - HA10	0.006747	0.016484	0.409282
HA16 - HA10	0.007870	0.019880	0.395865
HA17 - HA10	0.010153	0.023110	0.439341
HA18 - HA10	0.010153	0.026471	0.383568
HA19 - HA10	0.009012	0.019809	0.454937
HA20 - HA10	0.007878	0.016480	0.478031
HA21 - HA10	0.005637	0.013158	0.428401
HA22 - HA10	0.006752	0.013126	0.514433
HA23 - HA10	0.009020	0.013112	0.687955

Haplotype pair	K_a	K_s	K_a/K_s
HA1 - HA11	0.005626	0.029805	0.188773
HA2 - HA11	0.005643	0.036633	0.154050
HA3 - HA11	0.007887	0.036613	0.215427
HA4 - HA11	0.009009	0.029937	0.300931
HA5 - HA11	0.009009	0.036756	0.245108
HA6 - HA11	0.010141	0.036776	0.275749
HA7 - HA11	0.010175	0.016493	0.616943
HA8 - HA11	0.010146	0.026529	0.382434
HA9 - HA11	0.012412	0.019841	0.625569
HA10 - HA11	0.010148	0.023149	0.438369
HA12 - HA11	0.009024	0.019733	0.457290
HA13 - HA11	0.011288	0.016444	0.686468
HA14 - HA11	0.007886	0.019760	0.399092
HA15 - HA11	0.010144	0.019819	0.511812
HA16 - HA11	0.006741	0.023238	0.290080
HA17 - HA11	0.009019	0.026464	0.340818
HA18 - HA11	0.009019	0.029838	0.302273
HA19 - HA11	0.007880	0.023155	0.340314
HA20 - HA11	0.009011	0.019814	0.454769
HA21 - HA11	0.006770	0.016480	0.410818
HA22 - HA11	0.005623	0.016439	0.342069
HA23 - HA11	0.005625	0.016421	0.342572
HA1 - HA12	0.012447	0.023009	0.540968
HA2 - HA12	0.010199	0.029748	0.342863
HA3 - HA12	0.012445	0.029731	0.418578
HA4 - HA12	0.011290	0.023110	0.488526
HA5 - HA12	0.009018	0.029846	0.302161
HA6 - HA12	0.010151	0.029863	0.339936
HA7 - HA12	0.006778	0.009812	0.690760
HA8 - HA12	0.010156	0.019749	0.514272
HA9 - HA12	0.010151	0.013129	0.773112
HA10 - HA12	0.007889	0.016412	0.480676
HA11 - HA12	0.009024	0.019733	0.457290
HA13 - HA12	0.004499	0.003250	1.384329
HA14 - HA12	0.010165	0.013076	0.777380
HA15 - HA12	0.007886	0.013115	0.601286
HA16 - HA12	0.009011	0.016475	0.546934
HA17 - HA12	0.009029	0.019700	0.458301
HA18 - HA12	0.009029	0.023034	0.391961
HA19 - HA12	0.007888	0.016417	0.480500
HA20 - HA12	0.006755	0.013112	0.515189
HA21 - HA12	0.004511	0.009815	0.459644
HA22 - HA12	0.005629	0.009791	0.574939
HA23 - HA12	0.007896	0.009780	0.807309
HA1 - HA13	0.014722	0.019722	0.746466
HA2 - HA13	0.012475	0.026442	0.471810
HA3 - HA13	0.014719	0.026427	0.556959
HA4 - HA13	0.013558	0.019809	0.684472
HA5 - HA13	0.011282	0.026529	0.425248
HA6 - HA13	0.012417	0.026544	0.467780
HA7 - HA13	0.009044	0.006541	1.382580
HA8 - HA13	0.011285	0.016457	0.685710
HA9 - HA13	0.012416	0.009847	1.260858
HA10 - HA13	0.010151	0.013129	0.773112
HA11 - HA13	0.011288	0.016444	0.686468

Haplotype pair	K_a	K_s	K_a/K_s
HA12 - HA13	0.004499	0.003250	1.384329
HA14 - HA13	0.012433	0.009807	1.267820
HA15 - HA13	0.010147	0.009836	1.031568
HA16 - HA13	0.011272	0.013180	0.855245
HA17 - HA13	0.011294	0.016417	0.687986
HA18 - HA13	0.011294	0.019743	0.572056
HA19 - HA13	0.010150	0.013133	0.772828
HA20 - HA13	0.009013	0.009834	0.916594
HA21 - HA13	0.006772	0.006543	1.034989
HA22 - HA13	0.007887	0.006527	1.208294
HA23 - HA13	0.010159	0.006520	1.558146
HA1 - HA14	0.011302	0.023041	0.490512
HA2 - HA14	0.007913	0.029838	0.265186
HA3 - HA14	0.011300	0.029772	0.379535
HA4 - HA14	0.010149	0.023142	0.438531
HA5 - HA14	0.010149	0.029888	0.339558
HA6 - HA14	0.011283	0.029904	0.377292
HA7 - HA14	0.012467	0.009825	1.268869
HA8 - HA14	0.011288	0.019776	0.570793
HA9 - HA14	0.013558	0.013147	1.031261
HA10 - HA14	0.011290	0.016435	0.686974
HA11 - HA14	0.007886	0.019760	0.399092
HA12 - HA14	0.010165	0.013076	0.777380
HA13 - HA14	0.012433	0.009807	1.267820
HA15 - HA14	0.011286	0.013133	0.859347
HA16 - HA14	0.007875	0.016498	0.477326
HA17 - HA14	0.010160	0.019727	0.515030
HA18 - HA14	0.010160	0.023066	0.440477
HA19 - HA14	0.009018	0.016439	0.548547
HA20 - HA14	0.010151	0.013129	0.773112
HA21 - HA14	0.007909	0.009828	0.804722
HA22 - HA14	0.006757	0.009804	0.689184
HA23 - HA14	0.009026	0.009793	0.921648
HA1 - HA15	0.013569	0.023110	0.587120
HA2 - HA15	0.011324	0.029879	0.378995
HA3 - HA15	0.011288	0.029863	0.377992
HA4 - HA15	0.012410	0.023213	0.534613
HA5 - HA15	0.010138	0.026588	0.381302
HA6 - HA15	0.011271	0.026603	0.423674
HA7 - HA15	0.010174	0.009855	1.032408
HA8 - HA15	0.011276	0.019836	0.568480
HA9 - HA15	0.006742	0.013187	0.511226
HA10 - HA15	0.006747	0.016484	0.409282
HA11 - HA15	0.010144	0.019819	0.511812
HA12 - HA15	0.007886	0.013115	0.601286
HA13 - HA15	0.010147	0.009836	1.031568
HA14 - HA15	0.011286	0.013133	0.859347
HA16 - HA15	0.005611	0.016548	0.339056
HA17 - HA15	0.010150	0.019787	0.512947
HA18 - HA15	0.010150	0.023136	0.438693
HA19 - HA15	0.009008	0.016489	0.546330
HA20 - HA15	0.007875	0.013169	0.597979
HA21 - HA15	0.005635	0.009858	0.571620
HA22 - HA15	0.006750	0.009834	0.686411
HA23 - HA15	0.009017	0.009823	0.917940
HA1 - HA16	0.010144	0.026544	0.382151
HA2 - HA16	0.007901	0.033367	0.236808

Haplotype pair	K_a	K_s	K_a/K_s
HA3 - HA16	0.007876	0.033348	0.236185
HA4 - HA16	0.008996	0.026662	0.337425
HA5 - HA16	0.008996	0.030062	0.299259
HA6 - HA16	0.010127	0.030079	0.336669
HA7 - HA16	0.011303	0.013205	0.855933
HA8 - HA16	0.010131	0.023258	0.435619
HA9 - HA16	0.007864	0.016566	0.474690
HA10 - HA16	0.007870	0.019880	0.395865
HA11 - HA16	0.006741	0.023238	0.290080
HA12 - HA16	0.009011	0.016475	0.546934
HA13 - HA16	0.011272	0.013180	0.855245
HA14 - HA16	0.007875	0.016498	0.477326
HA15 - HA16	0.005611	0.016548	0.339056
HA17 - HA16	0.009007	0.023200	0.388217
HA18 - HA16	0.009007	0.026573	0.338931
HA19 - HA16	0.007869	0.019885	0.395718
HA20 - HA16	0.008998	0.016543	0.543915
HA21 - HA16	0.006761	0.013209	0.511821
HA22 - HA16	0.005615	0.013176	0.426173
HA23 - HA16	0.007876	0.013162	0.598420
HA1 - HA17	0.010164	0.029756	0.341575
HA2 - HA17	0.009050	0.036633	0.247040
HA3 - HA17	0.012439	0.036552	0.340310
HA4 - HA17	0.011285	0.029888	0.377572
HA5 - HA17	0.009014	0.036694	0.245656
HA6 - HA17	0.010147	0.036715	0.276366
HA7 - HA17	0.011325	0.016448	0.688547
HA8 - HA17	0.010151	0.026486	0.383284
HA9 - HA17	0.012419	0.019809	0.626958
HA10 - HA17	0.010153	0.023110	0.439341
HA11 - HA17	0.009019	0.026464	0.340818
HA12 - HA17	0.009029	0.019700	0.458301
HA13 - HA17	0.011294	0.016417	0.687986
HA14 - HA17	0.010160	0.019727	0.515030
HA15 - HA17	0.010150	0.019787	0.512947
HA16 - HA17	0.009007	0.023200	0.388217
HA18 - HA17	0.000000	0.003252	0.000000
HA19 - HA17	0.007884	0.023117	0.341069
HA20 - HA17	0.009016	0.019781	0.455777
HA21 - HA17	0.004509	0.013133	0.343346
HA22 - HA17	0.005626	0.016412	0.342824
HA23 - HA17	0.007892	0.016394	0.481383
HA1 - HA18	0.010164	0.033136	0.306731
HA2 - HA18	0.009050	0.040054	0.225942
HA3 - HA18	0.012439	0.039965	0.311248
HA4 - HA18	0.011285	0.026507	0.425721
HA5 - HA18	0.009014	0.040121	0.224675
HA6 - HA18	0.010147	0.040143	0.252762
HA7 - HA18	0.011325	0.019781	0.572521
HA8 - HA18	0.010151	0.029863	0.339936
HA9 - HA18	0.012419	0.023161	0.536198
HA10 - HA18	0.010153	0.026471	0.383568
HA11 - HA18	0.009019	0.029838	0.302273
HA12 - HA18	0.009029	0.023034	0.391961
HA13 - HA18	0.011294	0.019743	0.572056

Haplotype pair	K_a	K_s	K_a/K_s
HA14 - HA18	0.010160	0.023066	0.440477
HA15 - HA18	0.010150	0.023136	0.438693
HA16 - HA18	0.009007	0.026573	0.338931
HA17 - HA18	0.000000	0.003252	0.000000
HA19 - HA18	0.007884	0.026478	0.297771
HA20 - HA18	0.009016	0.02313	0.389799
HA21 - HA18	0.004509	0.009828	0.458802
HA22 - HA18	0.005626	0.019738	0.285056
HA23 - HA18	0.007892	0.019716	0.400269
HA1 - HA19	0.011293	0.026449	0.426982
HA2 - HA19	0.009048	0.033246	0.272155
HA3 - HA19	0.011291	0.033228	0.339808
HA4 - HA19	0.010141	0.026566	0.381726
HA5 - HA19	0.007876	0.033357	0.236097
HA6 - HA19	0.009006	0.033376	0.269827
HA7 - HA19	0.010177	0.013158	0.773456
HA8 - HA19	0.009010	0.023174	0.388792
HA9 - HA19	0.010705	0.018178	0.588931
HA10 - HA19	0.009012	0.019809	0.454937
HA11 - HA19	0.007880	0.023155	0.340314
HA12 - HA19	0.007888	0.016417	0.480500
HA13 - HA19	0.010150	0.013133	0.772828
HA14 - HA19	0.009018	0.016439	0.548547
HA15 - HA19	0.009008	0.016489	0.546330
HA16 - HA19	0.007869	0.019885	0.395718
HA17 - HA19	0.007884	0.023117	0.341069
HA18 - HA19	0.007884	0.026478	0.297771
HA20 - HA19	0.007877	0.016484	0.477855
HA21 - HA19	0.005636	0.013162	0.428244
HA22 - HA19	0.004494	0.013129	0.342314
HA23 - HA19	0.006754	0.013115	0.515000
HA1 - HA20	0.012430	0.023104	0.537984
HA2 - HA20	0.010185	0.029871	0.340964
HA3 - HA20	0.012427	0.029855	0.416260
HA4 - HA20	0.011274	0.023206	0.485824
HA5 - HA20	0.009006	0.029971	0.300485
HA6 - HA20	0.010137	0.029987	0.338049
HA7 - HA20	0.009038	0.009852	0.917339
HA8 - HA20	0.010142	0.019830	0.511434
HA9 - HA20	0.010136	0.013184	0.768856
HA10 - HA20	0.007878	0.016480	0.478031
HA11 - HA20	0.009011	0.019814	0.454769
HA12 - HA20	0.006755	0.013112	0.515189
HA13 - HA20	0.009013	0.009834	0.916594
HA14 - HA20	0.010151	0.013129	0.773112
HA15 - HA20	0.007875	0.013169	0.597979
HA16 - HA20	0.008998	0.016543	0.543915
HA17 - HA20	0.009016	0.019781	0.455777
HA18 - HA20	0.009016	0.023130	0.389799
HA19 - HA20	0.007877	0.016484	0.477855
HA21 - HA20	0.004505	0.009855	0.457120
HA22 - HA20	0.005621	0.009831	0.571789
HA23 - HA20	0.007884	0.009820	0.802887
HA1 - HA21	0.010188	0.019765	0.515448
HA2 - HA21	0.007936	0.026500	0.299464
HA3 - HA21	0.009046	0.026500	0.341342
HA4 - HA21	0.009035	0.013176	0.685732

Haplotype pair	K_a	K_s	K_a/K_s
HA5 - HA21	0.006766	0.026588	0.254486
HA6 - HA21	0.007899	0.026603	0.296904
HA7 - HA21	0.006791	0.006556	1.035839
HA8 - HA21	0.007902	0.016493	0.479120
HA9 - HA21	0.007898	0.009869	0.800297
HA10 - HA21	0.005637	0.013158	0.428401
HA11 - HA21	0.006770	0.016480	0.410818
HA12 - HA21	0.004511	0.009815	0.459644
HA13 - HA21	0.006772	0.006543	1.034989
HA14 - HA21	0.007909	0.009828	0.804722
HA15 - HA21	0.005635	0.009858	0.571620
HA16 - HA21	0.006761	0.013209	0.511821
HA17 - HA21	0.004509	0.013133	0.343346
HA18 - HA21	0.004509	0.009828	0.458802
HA19 - HA21	0.005636	0.013162	0.428244
HA20 - HA21	0.004505	0.009855	0.457120
HA22 - HA21	0.003379	0.006541	0.516516
HA23 - HA21	0.005642	0.006534	0.863421
HA1 - HA22	0.009026	0.019716	0.457795
HA2 - HA22	0.006780	0.026434	0.256472
HA3 - HA22	0.009024	0.026420	0.341574
HA4 - HA22	0.007880	0.019803	0.397917
HA5 - HA22	0.005620	0.026522	0.211903
HA6 - HA22	0.006748	0.026537	0.254286
HA7 - HA22	0.007908	0.006540	1.209286
HA8 - HA22	0.006751	0.016453	0.410338
HA9 - HA22	0.009010	0.009844	0.915249
HA10 - HA22	0.006752	0.013126	0.514433
HA11 - HA22	0.005623	0.016439	0.342069
HA12 - HA22	0.005629	0.009791	0.574939
HA13 - HA22	0.007887	0.006527	1.208294
HA14 - HA22	0.006757	0.009804	0.689184
HA15 - HA22	0.006750	0.009834	0.686411
HA16 - HA22	0.005615	0.013176	0.426173
HA17 - HA22	0.005626	0.016412	0.342824
HA18 - HA22	0.005626	0.019738	0.285056
HA19 - HA22	0.004494	0.013129	0.342314
HA20 - HA22	0.005621	0.009831	0.571789
HA22 - HA22	0.003379	0.006541	0.516516
HA23 - HA22	0.002246	0.000000	0.000000
HA1 - HA23	0.009029	0.019695	0.458469
HA2 - HA23	0.006782	0.026405	0.256852
HA3 - HA23	0.009028	0.026391	0.342079
HA4 - HA23	0.010151	0.019781	0.513136
HA5 - HA23	0.007883	0.026493	0.297551
HA6 - HA23	0.009014	0.026507	0.340063
HA7 - HA23	0.009045	0.006540	1.383087
HA8 - HA23	0.009018	0.016435	0.548749
HA9 - HA23	0.011284	0.009834	1.147474
HA10 - HA23	0.009020	0.013112	0.687955
HA11 - HA23	0.005625	0.016421	0.342572
HA12 - HA23	0.007896	0.009780	0.807309
HA13 - HA23	0.010159	0.006520	1.558146
HA14 - HA23	0.009026	0.009793	0.921648
HA15 - HA23	0.009017	0.009823	0.917940

Haplotype pair	K_a	K_s	K_a/K_s
HA16 - HA23	0.007876	0.013162	0.598420
HA17 - HA23	0.007892	0.016394	0.481383
HA18 - HA23	0.007892	0.019716	0.400269
HA19 - HA23	0.006754	0.013115	0.515000
HA20 - HA23	0.007884	0.009820	0.802887
HA21 - HA23	0.005642	0.006534	0.863421
HA22 - HA23	0.002246	0.000000	0.000000

Supplementary Table 8. Haplotypes of the ORF-A fragment of CHV-1 and their pairwise combinations that might have been under positive selection because of $K_a/K_s > 1$.

Haplotypes	Ka	Ks	Ka/Ks	Z score	P value
HA9#-HA7#	0.0124	0.0099	1.2619	16.3353	< 0.001***
HA13#-HA7#	0.0090	0.0065	1.3826	23.8637	< 0.001***
HA13#-HA9#	0.0124	0.0098	1.2609	16.2712	< 0.001***
HA13#-HA12#	0.0045	0.0033	1.3843	23.9728	< 0.001***
HA14#-HA7#	0.0125	0.0098	1.2689	16.7709	< 0.001***
HA14#-HA9#	0.0136	0.0131	1.0313	1.9498	0.0512^{ns}
HA14#-HA13#	0.0124	0.0098	1.2678	16.7055	< 0.001***
HA15-HA7#	0.0102	0.0099	1.0324	2.0214	0.0432*
HA15-HA13#	0.0101	0.0098	1.0316	1.9690	0.0489*
HA21-HA7#	0.0068	0.0066	1.0358	2.2355	0.0254*
HA21-HA13#	0.0068	0.0065	1.0350	2.1824	0.0291*
HA22-HA7#	0.0079	0.0065	1.2093	13.0543	< 0.001***
HA22-HA13#	0.0079	0.0065	1.2083	12.9924	< 0.001***
HA23-HA7#	0.0090	0.0065	1.3831	23.8953	< 0.001***
HA23-HA9#	0.0113	0.0098	1.1475	9.1988	< 0.001***
HA23-HA13#	0.0102	0.0065	1.5581	34.8148	< 0.001***

Variant of interest identified because of the presence of intolerant substitutions (through SIFT) and $K_a/K_s > 1$.

ns, not significant (p-value higher than 0.05); *, significant (p-value less than 0.05); ***, highly significant (p-value less than 0.001)

Supplementary Table S9. List of intolerant substitutions found in different haplotypes and Grantham's distance between the substituted amino acids of polyprotein ORF-A of CHV-1.

**Amino acid position**[a]	**Original amino acid**[b]	**New amino acid**[c]	**Grantham distance**[d]	**ORF-A haplotype**
186	A	T	58	HA14
221	E	K	56	HA10
222	Y	C	194*	HA9
268	P	T	38	HA20
285	R	C	180*	HA7
291	S	F	155*	HA4
301	S	N	46	HA7
307	V	M	21	HA11
400	S	L	145	HA9
435	A	V	64	HA19
480	G	E, R	98,125	HA4, HA9
481	T	S	58	HA13
485	L	F, V	22,32	HA9, HA19
496	V	T	69	HA10
509	L	F	22	HA4
533	L	S	145	HA14
552	V	A	64	HA13
553	Q	K	53	HA14
566	A	V	64	HA19
575	K	N	94	HA4
578	I	F	21	HA20

[a] Amino acid position as compared to EP721 (DQ861913)
[b] Original amino acid that was found most commonly in South Tyrol
[c] New amino acid resulting from the substitution in the codon
[d] Physico-chemical distance between mutated amino acids based on composition, polarity and molecular volume as explained by Grantham (1974)
* indicates radical change among amino acid based on Grantham's distance value as referred by Li et al. (1984)
